# Structured association analysis leads to insight into *Saccharomyces cerevisiae* gene regulation by finding multiple contributing eQTL hotspots associated with functional gene modules

**DOI:** 10.1186/1471-2164-14-196

**Published:** 2013-03-21

**Authors:** Ross E Curtis, Seyoung Kim, John L Woolford Jr, Wenjie Xu, Eric P Xing

**Affiliations:** 1 Joint Carnegie Mellon – University of Pittsburgh PhD Program in Computational Biology, Carnegie Mellon University, Pittsburgh, PAUSA; 2 Lane Center for Computational Biology, Carnegie Mellon University, Pittsburgh, PAUSA; 3 Department of Biological Sciences, Carnegie Mellon University, Pittsburgh, PAUSA; 4 Machine Learning Department, Carnegie Mellon University, Pittsburgh, PAUSA

## Abstract

**Background:**

Association analysis using genome-wide expression quantitative trait locus (eQTL) data investigates the effect that genetic variation has on cellular pathways and leads to the discovery of candidate regulators. Traditional analysis of eQTL data via pairwise statistical significance tests or linear regression does not leverage the availability of the structural information of the transcriptome, such as presence of gene networks that reveal correlation and potentially regulatory relationships among the study genes. We employ a new eQTL mapping algorithm, GFlasso, which we have previously developed for sparse structured regression, to reanalyze a genome-wide yeast dataset. GFlasso fully takes into account the dependencies among expression traits to suppress false positives and to enhance the signal/noise ratio. Thus, GFlasso leverages the gene-interaction network to discover the pleiotropic effects of genetic loci that perturb the expression level of multiple (rather than individual) genes, which enables us to gain more power in detecting previously neglected signals that are marginally weak but pleiotropically significant.

**Results:**

While eQTL hotspots in yeast have been reported previously as genomic regions controlling multiple genes, our analysis reveals additional novel eQTL hotspots and, more interestingly, uncovers groups of multiple contributing eQTL hotspots that affect the expression level of functional gene modules. To our knowledge, our study is the first to report this type of gene regulation stemming from multiple eQTL hotspots. Additionally, we report the results from in-depth bioinformatics analysis for three groups of these eQTL hotspots: ribosome biogenesis, telomere silencing, and retrotransposon biology. We suggest candidate regulators for the functional gene modules that map to each group of hotspots. Not only do we find that many of these candidate regulators contain mutations in the promoter and coding regions of the genes, in the case of the Ribi group, we provide experimental evidence suggesting that the identified candidates do regulate the target genes predicted by GFlasso.

**Conclusions:**

Thus, this structured association analysis of a yeast eQTL dataset via GFlasso, coupled with extensive bioinformatics analysis, discovers a novel regulation pattern between multiple eQTL hotspots and functional gene modules. Furthermore, this analysis demonstrates the potential of GFlasso as a powerful computational tool for eQTL studies that exploit the rich structural information among expression traits due to correlation, regulation, or other forms of biological dependencies.

## Background

Expression quantitative trait locus (eQTL) analysis has been widely used to understand how genetic variations in the genome perturb biological systems by altering cellular mRNA levels [1]. A typical eQTL study involves genotype data collected for genetic markers, such as single nucleotide polymorphisms (SNPs), along with microarray data for a population of individuals. These studies aim to identify genes whose expression level varies according to genetic variation. As both the genotype and gene expression data are collected at a genome-wide scale, *structured,* as opposed to the traditional trait-by-trait eQTL analysis must be employed to probe the complex interplay between the genome and phenome at a systems level rather than at the level of individual loci and genes. For example, genetic variation can perturb the expression of a gene, which then can affect the activity of other genes downstream in the same pathway. A mutation in a regulator, such as a transcription factor, can influence the expression of all of the regulator’s target genes, leading to a pleiotropic effect. On the other hand, genetic variants at multiple different loci may jointly influence the expression of some genes, through an additive or an epistatic effect. Analyzing a genome-wide eQTL dataset allows us to discover the complex patterns of how genetic variants give rise to variation in expression level. At the same time, examining multiple genes or multiple traits jointly in a genome-wide analysis can give insight into the functional roles that genetic variants play in a biological system and can potentially lead to the discovery of new regulators in a region of associated SNPs.

Many eQTL datasets have been collected for various organisms, including yeast [2], mouse [3], human [4], and Arabidopsis [5], as well as for different diseases such as diabetes [6]. Additionally, different statistical approaches have been developed that go beyond traditional single-trait analysis to unravel the complex patterns of association between the genetic variants and expression levels. In particular, we have previously developed a new statistical paradigm for eQTL mapping called structured association mapping [7]. In this paper we employ a structured association mapping method called GFlasso, which leverages the full gene-expression network to guide a search for genotypes that influence genes whose expression levels are highly correlated [7]. To our knowledge, this method is the first that systematically exploits the full gene correlation network in eQTL analysis.

GFlasso finds associations between SNPs and sub-networks of correlated genes within the full network by exploiting the gene network, avoiding many of the fundamental limitations of previous methods. For example, performing association analysis using the PCA-based method on transformed traits sacrificed the interpretability of the results. *Lirnet* used gene expression levels averaged over genes within each cluster and then maps these averages to genetic loci [8]. In this case, however, the averaging operation can lead to the loss of information on the activity of individual genes, especially genes whose expression levels are negatively correlated. Although Zhu et al. [9] and other work by the same group of researchers took advantage of the gene network in eQTL analyses, they did so only as a post-processing step after finding eQTLs for each gene separately, rather than directly using the network during the search for eQTLs. It is only recently that methods to exploit the rich information in the gene expression network have been developed [7,10-12].

In this study, we use structured association mapping to reanalyze the yeast eQTL dataset available from Brem and Kruglyak [13] with a new focus on uncovering the genetic basis behind the coupled gene-expression traits. The dataset includes the genome-wide profiling of expression levels and SNPs for 112 recombinant progeny from two parent strains, a laboratory strain and a wild vineyard strain. We choose this particular dataset because it has been previously analyzed using different computational methods, providing a useful test bed for comparing structured association mapping with other methods. Since the method we use, GFlasso, leverages the gene network in eQTL analysis to combine information across correlated traits, it has the potential to achieve greater statistical power and discover relatively weak association signals that were missed in previous analyses. In fact, our analysis of the yeast eQTL dataset stemming from GFlasso provided new insights into the complex interaction between genetic variations and the transcriptome in yeast.

Many of the previous computational analyses of this same dataset reported regions in the genome, coined eQTL hotspots, which control the expression level of gene clusters that are highly enriched for a common function [8,9]. This suggested a coordinated genetic control of gene modules. Also, by examining the eQTL hotspot regions in the genome, these studies identified candidate regulators whose genetic variations lead to a perturbation of the gene cluster’s gene expression levels.

While our structured association analysis rediscovers these previously reported eQTL hotspots and their regulators, we identify additional novel eQTL hotspots of biological significance. More interestingly, we find that many of these gene modules are associated with not one, but multiple eQTL hotspots. Although the presence of eQTL hotspots has been reported previously, to our knowledge our analysis is the first to find multiple eQTL hotspots that contribute to the same functional gene module. We perform in-depth bioinformatics analysis of three groups of these eQTL hotspots that we have uncovered. Based on the shared function of the genes perturbed by the eQTL hotspots in each group, we name the three groups the ribosome biogenesis (Ribi) group, the telomere group, and the retrotransposon group, and we suggest candidate regulators for each group of hotspots. Our bioinformatics analysis of each group of eQTL hotspots provides new insight into gene regulation in yeast. For example, in our analysis of the Ribi group, we find a coordinated regulation of ribosome biogenesis by multiple genomic loci on chromosomes II, V, and VII. We show experimentally that the expression levels of a subset of 43 of these target genes change when the *PBF2*, *KAP114*, *RAI1*, and *REI1* are knocked out. In our analysis of the telomere group, we discover candidate regulators (*NUP2*, *RIF2*, *SIR3*, *YRF1*) in four genomic regions on chromosome IV, X, and XII that likely play a coordinated role in telomere silencing. We identify mutations in the promoter and coding regions of the candidate regulators using the full genome sequences available from public databases to provide strong evidence that the candidate regulators are true regulators. Finally, in the retrotransposon group, we discover the coordinated effects of 17 retrotransposon insertions on the resulting expression signal for retrotransposons. We note that in all three modules, this coordinated effect could be epistatic or additive.

## Results

We applied GFlasso to analyze a genome-wide eQTL dataset generated from a cross between the BY4617 (BY) strain (isogenic to yeast strain S288c) and the vineyard RM11-1a (RM) strain of *Saccharomyces cerevisiae*, baker's yeast [13]. The dataset consists of these two parent strains and 112 recombinant progeny. We considered the 1260 unique SNP markers on all 16 chromosomes, which cover nearly the entire genome at a resolution of about 20 kb. We used these SNP markers to find associations to the mRNA expression levels for 5637 genes (genes with more than 30% missing values were excluded from analysis).

GFlasso assumes that a network for the gene-expression traits is available as prior knowledge, and GFlasso leverages this network in a structured sparse regression framework to identify associations between genetic loci and multiple traits that are tightly connected in the network. In our preprocessing step, we constructed a scale-free and modular network from the gene-expression data (see Methods for more detail) [9,14]. We used the resulting topological overlap matrix [14] as our gene-expression network. Once we estimated the parameters for association strengths using GFlasso (see Methods), we carried out an in-depth biological analysis. We have automated the full pipeline of our analysis and made it available with our distribution of the GenAMap software platform for genome-wide association (GWAS) and eQTL analysis [15,16].

### Gene modules under regulation of common genetic loci

**Figure 1:**
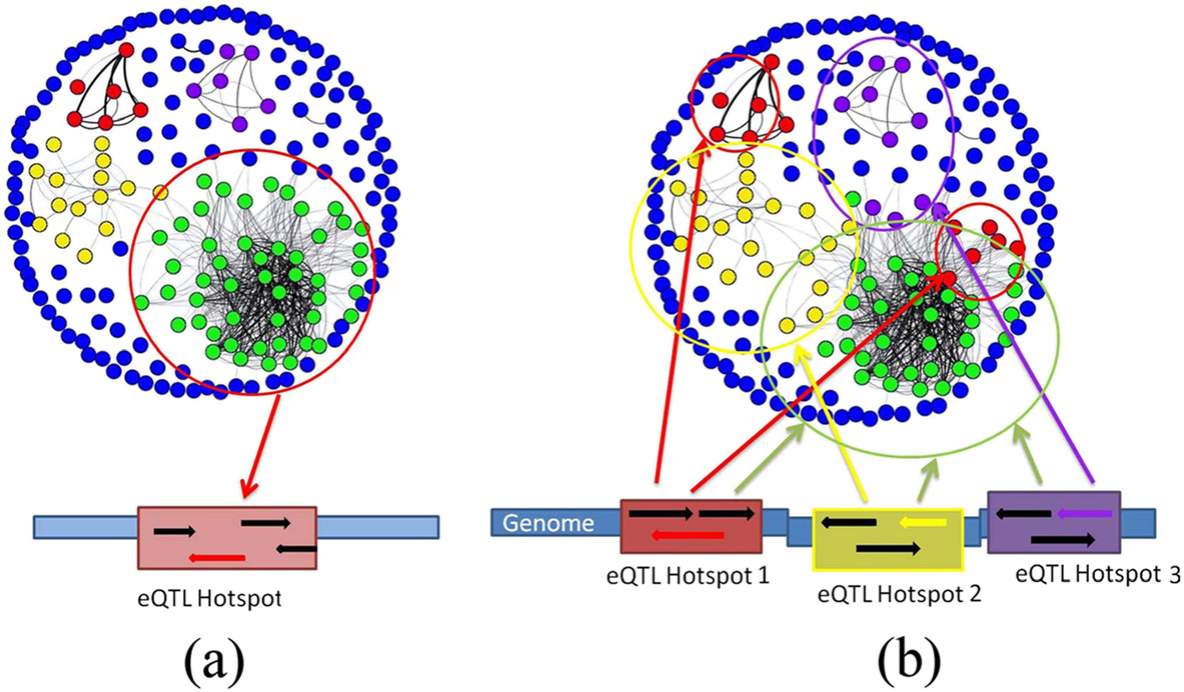
**An illustration of our main results.** Figure legend text (**a**) Previous analyses (e.g., [9,17,35]) of the yeast eQTL dataset reported eQTL hotspots, a module of multiple genes controlled by the same genomic locus. (**b**) In our GFlasso analysis of the same dataset, we not only found eQTL hotspots, but also discovered multiple contributing eQTL hotspots, where the same module of multiple genes is associated with multiple eQTL hotspots. This figure was created using GenAMap **[**[16]**]**.

We examined the eQTLs estimated by GFlasso for clusters of genes controlled by common genetic loci (Figure 1). We divided up the genome into 428 genomic regions based on the linkage disequilibrium (LD) between the SNPs in this dataset (see in Additional file 1: Supplementary Methods). We define an *eQTL module* as all of the genes that map to the same genomic region. We define an *eQTL hotspot* as a genomic region whose eQTL module is greater than 40 genes. An eQTL hotspot and its corresponding eQTL module imply a pleiotropic effect of the genetic locus on co-regulated genes in a common pathway. We note that in our definition of an eQTL module, a gene could be a member of multiple eQTL modules, each associated with different eQTL hotspots. In a biological system, this corresponds to the situation of multiple contributing genetic loci, where the expression of the gene is affected by multiple *trans*-acting loci as well as a possible *cis*-acting locus through either an additive or an epistatic effect.

Although we found many eQTL modules, in this study we focus on those eQTL modules with greater than 40 genes, that is, only those that map to eQTL hotspots (in Additional file 1: Figure S1). We present an analysis of 22 such eQTL modules that vary in size from 42 to 722 genes. Ten of the 22 corresponding eQTL hotspots were novel discoveries in this dataset. The other 12 eQTL hotspots overlapped with the 13 eQTL hotspots that had been reported in previous analyses of the same dataset; all 13 previous eQTL hotspots were recovered as two previously discovered eQTL hotspots were combined in our analysis [8,9,17].

The common association that an eQTL hotspot has to all the genes in an eQTL module suggests that the region harbors regulators that influence the expression levels of the genes in the eQTL module. We list some candidate regulators located in *cis* to each eQTL hotspot in Table 1. All the genes within 20 kb from the eQTL hotspot are potential candidates, but because many genes are located in *cis* to each eQTL hotspot, we select transcription factors, genes in the eQTL module, and other genes involved in the pathway of the eQTL module to list here. In Table 1, we compare our results with those obtained from a computational analysis using a Bayesian network modeling approach [9], *Lirnet*[8], and known and possible regulators based on literature search [17]. In general, we found that the results were consistent between GFlasso and previous analyses. For example, in eQTL hotspot 4 located on chromosome III around 200 kb, GFlasso found 62 genes in the eQTL module; five of these genes, *MATALPHA1*, *MATALHPA2*, *PHO87*, *BUD5*, and *TAF2*, are located in *cis* to this eQTL hotspot and therefore they are candidate regulators of the eQTL module. Consistent with our results, three previous analyses discussed *MATALPHA1* as a regulator for genes in this eQTL module, and *Lirnet* additionally suggested *MATALPHA2* and *TBK1*. As these candidate regulators lie in *cis* to this eQTL hotspot region, the genetic variation in this region may directly influence the activity or expression of these regulators, which then influence the expression of other genes in the eQTL module.

**Table 1:** The eQTL hotspots and their candidate regulators from GFlasso and other previous analyses

**eQTL Hotspot**	**eQTL module size**	***cis *genes in eQTL module GFlasso**	**Yvert et al. [**[17]**]**	**Zhu et al. [**[9]**]**	**Lee et al. [**[8]**]**
*II:380000	106	*NRG1 TIP1 TAT1 TEC1 ECM33*	none	none	*RDH54 SEC18 SPT7*
*II:560000	722	*AMN1 MAK5 CNS1 TBs1 TOS1 ARA1 SUP45 CSH1 RPB5 SDS24 ENP1 REI1*	*AMN1 MAK5*	*AMN1 CNS1 TBS1 TOS1 ARA1 SUP45 CSH1*	*AMN1 CNS1 TOS2 ABD1 PRP5 TRS20*
*III:100000	225	*LEU2 ILV6 NFS1 CIT2 PGS1 RER1 HIS4 FRM2 KCC4*	*LEU2*	*LEU2 ILV6 NFS1 CIT2 MATALPHA1*	*LEU2 ILV6 PGS1*
*III:200000	62	*MATALPHA1 MATALPHA2 PHO87 BUD5 TAF2*	*MATALPHA1*	*MATALPHA1*	*MATALPHA1 MATALPHA2 TBK1*
**IV:1500000**	46	*YRF1-1 YDR539W YDR541C*	-	-	-
*V:110000	45	*URA3 NPP2*	*URA3*	*URA3*	*URA3 NPP2 PAC2*
**V:350000**	618	*RPS24A RPS8B RTT105*	-	-	-
**V:420000**	405	*LCP5 NSA2*	-	-	-
**V:460000**	42	*YER138C UBP5 RTR1*	-	-	-
**VII:50000**	350	*RAI1 TAD1 KAP114*	-	-	-
*VIII:110000	147	*GPA1 YAP3 ERG11 LAG1 SHO1 ETP1 YLF2 LEU5*	*GPA1*	*GPA1*	*GPA1 STP2 NEM1*
**X:20000**	48	*YJL225C VTH2 FSP2 REE1*	-	-	-
**XII:610000**	53	*TOP3*	-	-	-
*XII:680000	185	*HAP1 NEJ1 SSP120*	*HAP1*	*HAP1*	*HAP1 NEJ1 GSY2*
**XII:780000**	44	*REC102 PEX30 FKS1 GAS2*	-	-	-
*XII:1070000	54	*YRF1-4 YRF1-5 YLR464W YLR462W*	*SIR3*	*YRF1-4 YRF1-5 YLR464W*	*SIR3 HMG2 ECM7*
*XII:70000	76	*MDM1*	none	none	*ARG81 TAF13 CAC2*
*XIV:490000	448	*SAL1 TOP2 MKT1 THO2 MSK1 TPM1 LAT1 SWS2*	none	*SAL1 TOP2*	*TOP2 MKT1 MSK1*
**XIV:550000**	45	*COG6 YIP3 HDA1*	-	-	-
**XV:90000**	85	*ZEO1 RFC4 HM11 INO4 NDJ1 SKM1 HAL9*	-	-	-
*XV:180000	406	*PHM7 ATG19 WRS1 RFC4*	none	*PHM7*	*PHM7 ATG19 BRX1*
*XV:59000	120	*LSC1 YOR131C RAS1 INP53 OST2 PIN2*	*CAT5*	none	*CAT5 ADE2 ORT1*

* denotes a previously discovered hotspot.

### Multiple contributing eQTL hotspots with pleiotropic effects on common eQTL modules

In order to see if the genes in an eQTL module controlled by an eQTL hotspot share function, we performed a gene ontology (GO) enrichment analysis using the GO Slim annotation from the *Saccharomyces* Genome Database (SGD) [18]. As can be seen in Table 2, many of the eQTL modules were significantly enriched for common GO categories, indicating the genes in the eQTL module share function. For example, the eQTL module associated with the eQTL hotspot at XII:680 kb is enriched for genes annotated to lipid metabolic process (GO category, *p-*value = 1.3e-14) in biological process (GO category type), and the eQTL module associated with the XIV:450 kb eQTL hotspot is enriched for translation (GO Category, *p-*value = 2.3e-28) in biological process (GO category type). For the previously reported eQTL hotspots, our results were consistent with those from previous GO enrichment analysis.

**Table 2:** GO enrichment analysis of eQTL modules/hotspots found by GFlasso

**Group**	**eQTL Hotspot**	**eQTL module size**	**GO Category**	***p*-value**	**GO size (overlap)**	**GO Annotation [**[9]**]**
**Ribi Group**	*II:560000	722	nucleolus	1.4e-81	224 (148)	Cytoplasm organization and biogenesis
			ribosome biogenesis	5.4e-62	311 (156)	
	V:350000	618	ribosome biogenesis	7.0e-104	311 (184)	
			nucleolus	5.5e-89	224 (129)	
	V:420000	405	nucleolus	8.0e-80	224 (118)	
			Ribosome biogenesis	6.8e-66	311 (124)	
	VII:50000	350	nucleolus	7.6e-97	224 (124)	
			ribosome biogenesis	5.6e-83	311 (131)	
**Telomere Group**	IV:150000	46	cellular component unknown	6.1e-21	683 (33)	
			helicase activity	1.8e-17	80 (15)	
	X:20000	48	cellular component unknown	1.6e-22	683 (35)	
			helicase activity	1.4e-15	80 (14)	
	XII:780000	44	helicase activity	8.3e-10	80 (15)	
			cellular component unknown	1.2e-13	683 (26)	
	*XII:1070000	54	helicase activity	9.3e-19	80 (15)	none
			cellular component unknown	1.1e-16	683 (27)	
**Retrotransposon Group**	V:460000	42	none	-	-	
	*VIII:110000	147	conjugation site of polarized growth	1.6e-11	119 (20)	conjugation, RNA binding
				7.6e-6	211 (18)	
	XV:90000	85	none	-	-	
**Other eQTL modules**	*II:380000	106	none	-	-	
	*III:100000	168	cell. amino acid derivative proc	6.3e-31	215 (55)	Organic acid metabolism
			transferase activity	2.6e-7	623 (51)	
	*III:200000	62	conjugation	5.1e-7	119 (10)	Response to chemical stimulus
			response to chem stimulus	4.3e-6	400 (16)	
	*V:110000	45	carbohydrate met. process	1.2e-4	249 (9)	
			cell. aromatic comp. met proc	1.5e-4	65 (5)	
	XII:610000	53	none	-	-	
	*XII:680000	185	lipid metabolic process	1.3e-14	249 (36)	Lipid metabolism, ER
			ER	3.9e-10	350 (36)	
	*XIII:70000	76	cell. amino acid derivative proc	1.2e-4	215 (11)	
	*XIV:450000	448	translation structural molecule activity	2.3e-28	373 (97)	Protein biosynthesis, intracellular transport
				1.4e-15	325 (7)	
	XIV:550000	45	none	-	-	
	*XV:180000	406	carbohydrate met. process	3.5e-5	249 (36)	Gen of precursor met & energy
			protein modification process	4.8e-4	544 (21)	
	*XV:590000	76	gen of precursor met. & energy	2.6e-35	168 (42)	Gen of precursor met & energy
			mitochondrial envelope	3.8e-20	82 (37)	

* denotes a previously discovered hotspot.

In addition, we performed enrichment analyses on each of the eQTL modules using two knockout datasets [19,20] and four transcription factor binding datasets [21-24]. The results from these enrichment analyses are available in the Additional file 1: Supplementary Methods and the Additional file 2: Supplementary GFlasso Results. From the transcription factor target enrichment results, we found that genes in each eQTL module involved in the same GO process were also generally enriched for the binding of a common transcription factor and a knockout perturbation as has been shown before [9].

**Figure 2:**
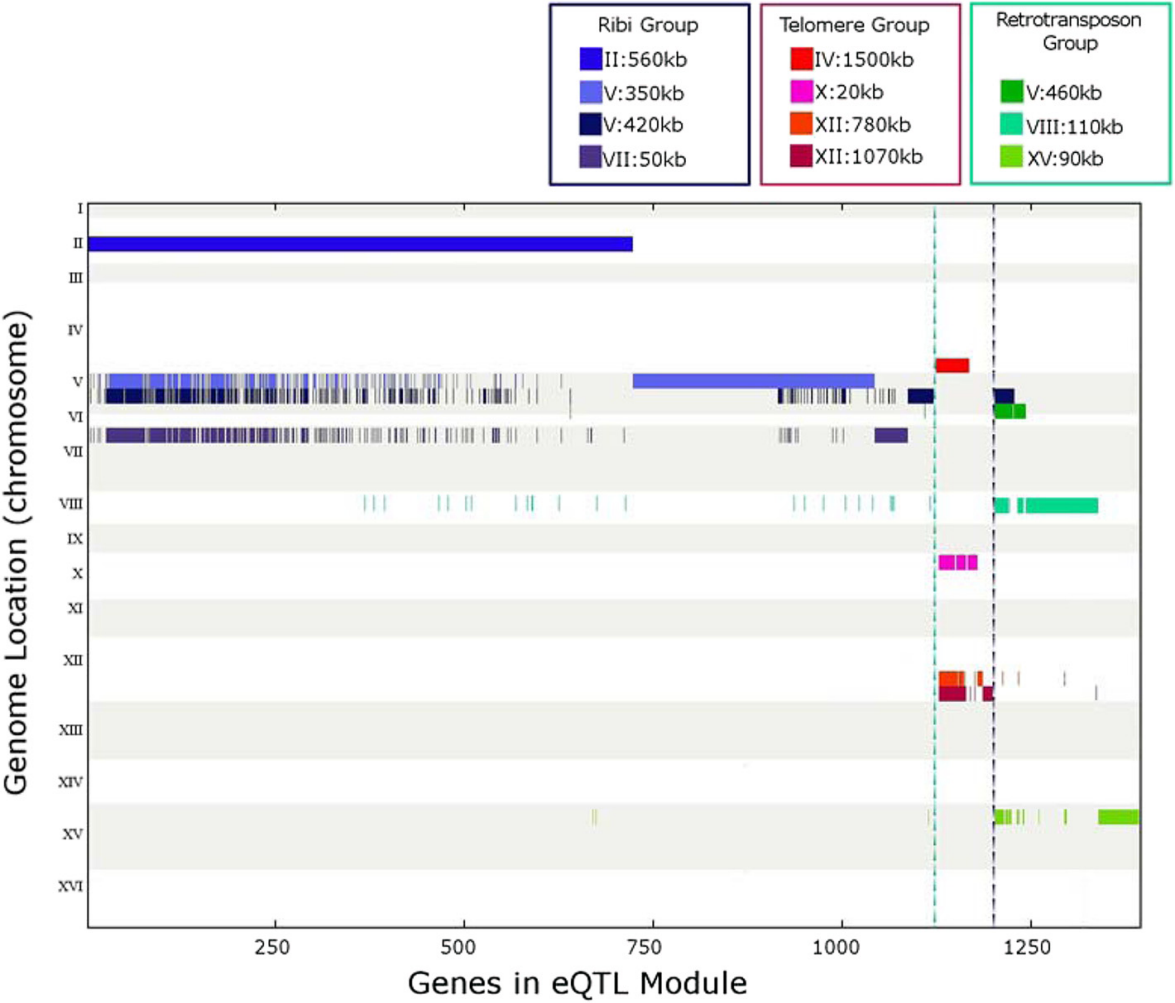
**eQTL hotspots and their overlapping eQTL modules found by GFlasso.** GFlasso found that many genes (*x*-axis) are jointly influenced by the same genetic loci (*y*-axis), suggesting that these eQTL hotspots perturb an overlapping set of genes. We group these eQTL hotspots into the Ribi, telomere, and retrotransposon groups according to their overlaps in the corresponding eQTL modules. For example, the first 722 genes (plotted along the *x*-axis) all belong to the eQTL module for II:560 kb, and many of these genes also belong to the eQTL modules derived from three other eQTL hotspots: V:350 kb, V:420 kb, and VII:50 kb.

**Figure 3:**
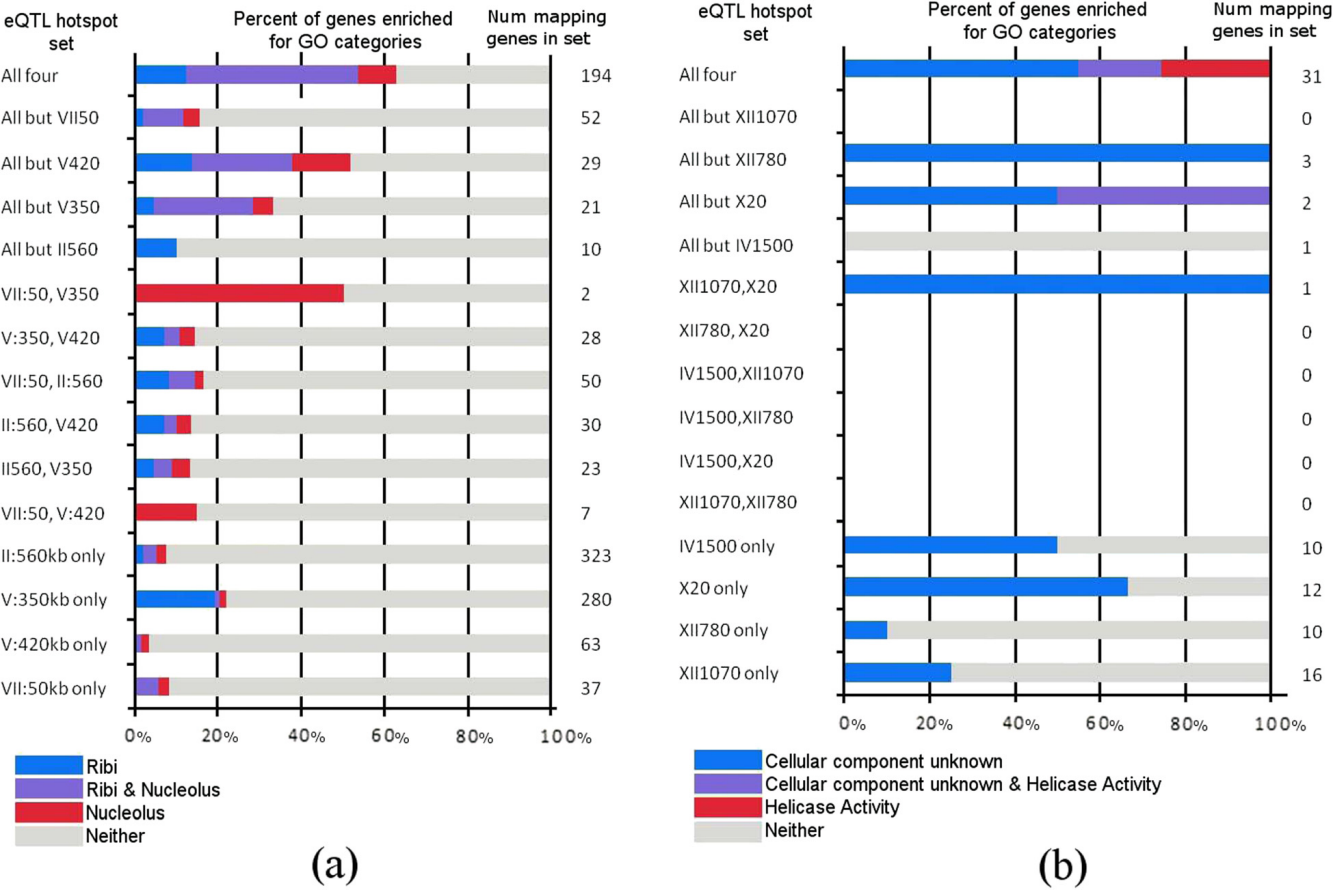
**GO enrichment analysis for eQTL hotspots with overlapping eQTL modules.** For both the (**a**) Ribi group and the (**b**) Telomere group, we divide all of the genes in the overlapping eQTL modules into different sets (rows) based on which of the eQTL hotspots in each eQTL hotspot group they are mapped to. Genes that map to all four eQTL hotspots are placed in the set labeled “All four” in the top row. Within each of the sets, we show the percentage of genes mapping to the eQTL hotspot(s) that are annotated to one or both of the top GO enrichment categories for the eQTL hotspot group’s eQTL modules along the *x* axis with different colors. In our analysis, we focus our attention on the genes with associations to three or more eQTL hotspots in a group, as they are enriched for a common function.

Interestingly, as we considered the results from the GO enrichment analysis and the transcription factor and knockout analyses, we noticed groups of eQTL modules that were enriched for the same GO annotations, transcription factor binding, and knockout signatures. For example, the eQTL modules associated to eQTL hotspots II:560 kb, V:350 kb, V:420 kb, and VII:50 kb were all significantly enriched for ribosome biogenesis and *PBF2* binding. Furthermore, as shown in Figure 2, these four eQTL modules had a large overlap in member genes. This suggests that a large number of genes in the four eQTL modules are regulated in-part by each of these different eQTL hotspots. As the eQTL modules share the common GO annotation of ribosome biogenesis (Ribi), we call this group of eQTL hotspots the Ribi group (Figure 3).

Additionally, we found another group of eQTL modules, associated with eQTL hotspots IV:1500 kb, X:20 kb, XII:780 kb, and XII:1070 kb, all significantly enriched for helicase activity. Again, the eQTL modules corresponding to these eQTL hotspots shared a large fraction of member genes (Figure 2), and many of the genes that are shared by these four eQTL modules were annotated with common GO terms (Figure 3). Since our bioinformatics analysis of this group of eQTL modules revealed that they are involved in telomere activity, we name this group of eQTL hotspots the telomere group. As we considered the telomere group and the Ribi group, we noticed that the genes regulated by three or more of the eQTL hotspots in the group had a higher GO enrichment than genes that were associated with only one or two eQTL hotspots (Figure 3). Thus, in subsequent analysis we focus primarily on the genes associated with at least three of the four eQTL hotspots.

In addition to the Ribi and telomere groups of eQTL hotspots, we searched for other groups of eQTL hotspots with an overlap of more than 20 genes in the corresponding eQTL modules. Using this criterion, we identified one additional group of eQTL modules mapping to eQTL hotspots at V:460 kb, VIII:110 kb, and XV:90 kb (Table 2). Although we did not find a common GO enrichment for these eQTL modules, further analysis revealed that many of the genes are involved in retrotransposon biology. Thus, we name this group the retrotransposon group.

We note that only one of the eQTL hotspots associated with each of the Ribi, telomere, and retrotransposon groups has been found in previous analyses of the same dataset, while all of the other eQTL hotspots associated to the groups of eQTL modules are novel discoveries from our GFlasso analysis. Thus, to our knowledge, these groups of eQTL hotspots with a common eQTL module have not been found in any of the previous analyses of this dataset. Thus, our GFlasso and bioinformatics analysis provides new insight into the genetic control of gene expression in a cell, especially the pleiotropic effect of multiple contributing genetic loci.

We compared the GFlasso results to those from a previous study which looked for epistatic interactions in this dataset [25]. After replicating the previous analysis, we found few overlaps between the results from the two analyses; the two studies share only one gene affected by the same two genomic bins (see in Additional file 1: Figure S2). The differences in the two results sets demonstrate the different characteristics of the two methods used in the analyses. For example, GFlasso is built off of an additive model, and thus will pick up on regulation signals that will not be discovered in epistatic analyses. Furthermore, GFlasso considers the whole gene-expression network to find associations between SNPs and a group of genes with highly correlated expression levels. Meanwhile, the analysis in Brem et al. [25] examined expression for each gene individually. As GFlasso tends to focus on pleiotropic effects by combining information across multiple genes in the gene network, it discovered the additive effect of multiple genomic regions on gene modules. On the other hand, these signals were missed in the analysis by Brem et a.l [25], which was looking for epistatic interactions among loci with effects on individual genes.

Another recent study looked at the pleiotropic and epistatic effects among eQTL modules in this dataset [26]. In that study, Zhang et al. used a Bayesian framework to look for associations between genomic regions and modules of genes. This approach varies significantly from GFlasso in its underlying assumptions and model, especially in reference to its focus on epistatic effects. While the approaches differ, the two models are complimentary in the results that the find. For example, Zhang et al. report multiple gene modules associated with approximately 21 different genomic regions. Of these 21 genomic regions, all but two are in close proximity to the genomic regions found by GFlasso. The modules from Zhang et al.'s paper generally include fewer genes than the modules found by GFlasso and often have similar annotations. However, GFlasso finds an additional 12 genomic regions associated with an eQTL module that are not found by Zhang et al. Three regions (V:350 kb, V:420 kb, and VII:50 kb) from the Ribi group, two regions (XII:780 kb, 1070 kb) from the Telomere group, and two regions (V:460 kb, XV:90 kb) from the Retrotransposon group are found by GFlasso but not by Zhang et al. Another important difference between the methods is that while we create our eQTL modules based off of the association of multiple genes to one genomic region, Zhang et al. find multiple gene modules associated to a single genomic region. If using GFlasso, one could analyze eQTL modules using a network analysis approach to obtain a similar result.

### Multiple genes in three eQTL hotspots affect Ribi expression levels

Given the evidence of multiple contributing eQTL hotspots from the GFlasso and enrichment analyses, we performed bioinformatics analysis on the three groups of eQTL hotspots: the Ribi, telomere, and retrotransposon groups. We determined the functional role that these contributing loci play on the overlapping set of genes and identified potential candidate regulators in *cis* to these loci. By further examining the DNA sequence of these potential candidate regulators, we found that many of them have missense or promoter mutations between the two strains. The presence of such mutations indicates a potential change of function or expression level. In the case of the Ribi group, we further verify some of the regulators experimentally. Below, we present our analysis of the Ribi, telomere, and retrotransposon groups.

We first consider the Ribi group, which consists of four eQTL hotspots located on chromosomes II:560 kb, V:350 kb, V:420 kb, and VII:50 kb. The corresponding eQTL modules overlap, with 194 genes in the overlap. 122 of these 194 genes have the GO annotation for ribosome biogenesis (GO category, type of biological function) and/or nucleolus (GO category, type of cellular compartment).

In our analysis of these four eQTL hotspots, we found evidence of direct and indirect regulation in the Ribi regulation system and identified previously unknown potential regulators, which were confirmed through experimental validation. The creation of a Ribi protein is a multi-step process, and there are many steps along the pathway where transcriptional feedback could potentially occur. First the genes encoding the RPs (ribosomal proteins) and Ribi assembly factors must be transcribed, the transcripts translated, and then the proteins imported into the nucleus where the Ribi assembly factors assemble rRNA with the RPs into functional ribosomes. Thus, expression levels of important genes can affect the expression of genes directly, or through indirect feedback loops during any step of this process.

**Figure 4:**
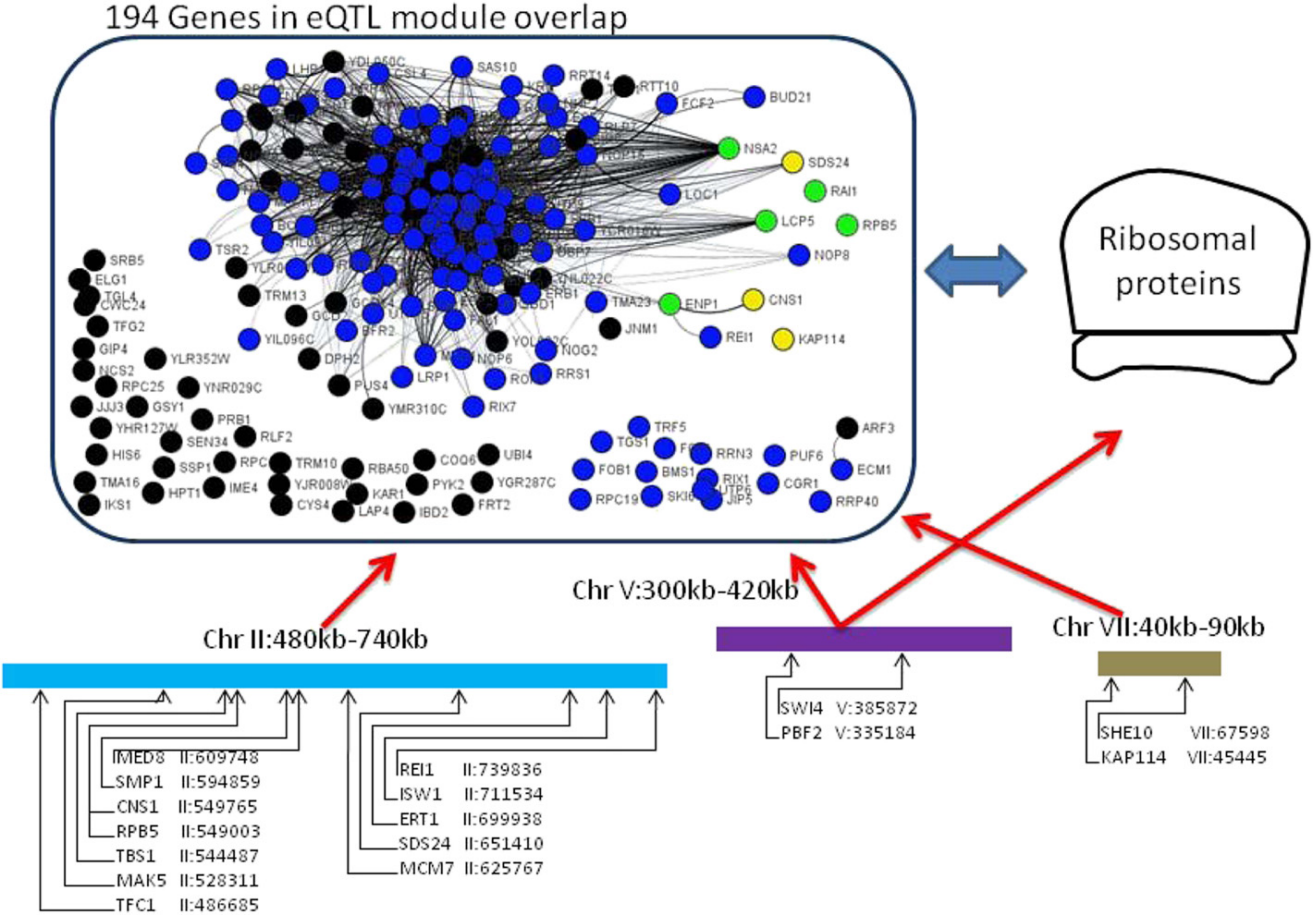
**An illustration of gene regulation in the Ribi group of eQTL hotspots.** We found four eQTL hotspots on chromosomes II, V, and VII that are all associated with the same 194 genes. 122 of the genes in this 194-gene overlap were annotated to the GO category of ribosome biogenesis (Ribi) or nucleolus (shown as blue nodes in the graph). The genes involved in Ribi are generally assembly factors that assemble rRNA and ribosomal proteins into the ribosomal unit in the nucleus. We also found an association from the V:350 kb eQTL hotspot to the ribosomal proteins. The expression levels of the ribosomal proteins are tightly coupled with the expression of the Ribi genes. Additionally, we found eight genes (shown as green and yellow nodes in the graph) in the overlap that were located in *cis* to one of these eQTL hotspots. The green nodes represent genes located in *cis* that are annotated for the Ribi or nucleolus GO categories, while the yellow nodes represent genes located in *cis* to one of the eQTL hotspots with a different GO annotation (see Table 3). This figure was created using GenAMap.

To limit our search for candidate regulators in *cis* to the four eQTL hotspots in the Ribi group, we considered genes that were located in *cis* to one of the four eQTL hotspots and either 1) were also found in the 194 gene overlap, implying an association to all four hotspots (8 overall, shown as green and yellow nodes in Figure 4), 2) were also known to be involved in Ribi (*MAK5*, *UTP7*, and *PBF2*), or 3) were annotated as a DNA binding protein (12 overall, listed as DNA binding in Table 3). We list the candidate genes from our search in Table 3. Also, in Figure 4 we provide a visual overview of the regulation of these eQTL modules. Although there might be other genes in *cis* to these eQTL hotspots that could affect expression levels of the genes in these eQTL modules, we believe our criteria for candidate regulators led us to many of the interesting possibilities.

**Table 3:** Candidate regulators in the Ribi group of eQTL hotspots

**eQTL Hotspot**	**Candidate**	**GO Category**	**Differentially Expressed?**	**Mutations**	**Function**
**II:560 kb**	TFC1	DNA binding	No	3 missense	RNA Pol III subunit
	MAK5	Ribi	No	5 missense, 4 indel	60S ribosome processing
	TBS1	DNA binding	6.7e-20	7 missense	Unknown
	RPB5	Nucleolus	9.6e-3	promoter has 7 SNPs and 1 indel	RNA Poly subunit
	CNS1	Protein folding	3.6e-9	promoter has 7 SNPs and 1 indel, 1 missense	TPR-containing co-chaperone
	SMP1	DNA binding	2.7e-2	promoter has 7 SNPs and 2 indels	Transcription factor that regulates osmotic stress
	MED8	DNA binding	1.2e-2	Promoter has 2 SNPs, 2 missense	RNA Poly II mediator complex
	MCM7	DNA binding	No	6 missense	DNA ATPase activity
	SDS24	Molecular function unknown	7.2e-7	2 missense, 2 promoter SNPs	Involved in cell separation during budding
	ERT1	DNA binding	2.2e-2	5 missense, 8 SNPs in promoter and a 5 base insertion	Transcriptional regulator of nonfermentable carbon utilization
	THI2	DNA binding	No	None	Zinc finger protein
	ENP1	Ribi	No	None	40S ribosomal subunit synthesis
	ISW1	DNA binding	No	1 missense	ATPase, DNA and nucleosome binding
	REI1	Ribi	No	3 missense	Cytoplasmic pre-60S factor
**V:350 kb**	UTP7	Ribi	No	None	Processing of 18S rRNA
	RAD51	DNA binding	No	None	Strand exchange protein
	PBF2	Ribi	3.0e-2	9 missense	PAC binding factor
	SWI4	DNA binding	No	2 missense	Transcriptional activator
**V:420 kb**	NSA2	Ribi	1.4e-5	None	Constituent of 60S pre-ribosomal particles
	LCP5	Ribi	2.6e-3	1 missense	Involved in maturation of 18S rRNA
	YER130C	DNA binding	No	1 missense	Unknown function
**VII:70 kb**	RAI1	Ribi	No	None	Required for pre-rRNA processing
	RTF1	DNA binding	No	None	Subunit of RNA Pol II
	KAP114	Protein import into nucleus	1.2e-8	9 missense	Karyopherin

In both this analysis and the telomere analysis, we examine our candidate regulators by comparing the full coding and promoter sequence for the BY and RM strains. Since only 1260 unique (2956 overall) SNPs were genotyped in the eQTL dataset, these SNPs serve only as genetic markers rather than an exhaustive list of genetic polymorphisms between the two strains. Once the GFlasso analysis points us to the genomic regions or eQTL hotspot around a genetic marker, we can compare the full sequences available from public databases (see Methods) to identify missense and promoter mutations.

### Validating discovered Ribi regulator genes experimentally

**Figure 5:**
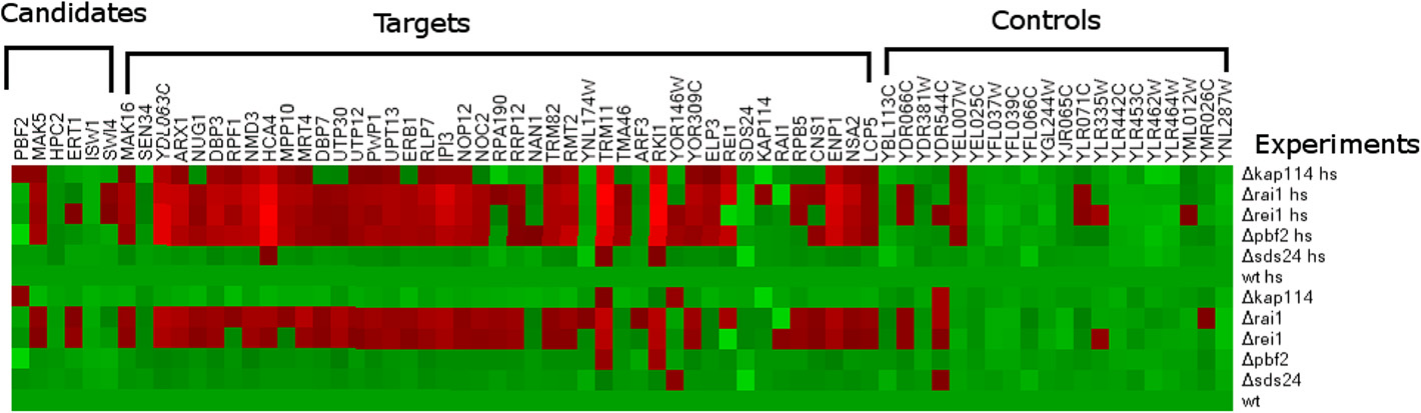
**Knockout experiment results.** We show the results from the knockout experiments using a graphical representation of the data. This plot was generated with Heatmap Builder [48] using the log values of the normalized gene expression measurements. The red pixels in the graph represent an over-expression of the genes in the experiment, while the bright green pixels represent under-expression. These results suggest that GFlasso correctly identified target genes that change expression due to mutations in the eQTL hotspots that were identified.

Based on our mutation analysis, we selected five candidate regulators from three of the four eQTL hotspots for further validation using knock-out experiments (*PBF2, SDS24, REI1, RAI1,* and *KAP114*). We did not select candidates from the V:420 kb hotspot because our candidates in this region are essential genes. For each candidate regulator that we selected, we grew a knock-out strain under normal and heat shock conditions, and then compared normalized expression levels of 43 targets (from the GFlasso results) with the expression levels from the wild-type (see Methods). We consider a gene to be affected by the knock-out if its expression is more than 1.5 times greater than wild-type (or if wild-type level is more than 1.5 times greater than the knock-out). A summary of the results is available in Table 4, and a spreadsheet of the experimental results is available in the Additional file 3. In Table 4, we report the percent of the target genes affected by the knock-out, in addition to the average and maximum fold change when compared to the wild type. These summary statistics help to demonstrate the effect that knocking out each candidate has on the targets predicted by GFlasso. We also include a pictorial representation of the results in Figure 5, showing which target genes were overexpressed in the different experiments.

**Table 4:** Summary of knock-out results for candidate regulators in the Ribi group

	***ΔSDS24***	***ΔPBF2***	***ΔREI1***	***ΔRAI1***	***ΔKAP114***	***ΔSDS24 *HS**	***ΔPBF2 *HS**	***ΔREI1 *HS**	***ΔRAI1 *HS**	***ΔKAP114 *HS**
% targets affected	4.7%	4.7%	74.4%	83.7%	4.7%	7.0%	79.1%	79.1%	86.0%	60.5%
max change	1.691	1.983	2.686	2.934	1.838	1.531	5.117	5.700	5.977	3.847
avg. change	1.168	1.245	1.641	1.824	0.962	1.174	2.206	2.241	2.417	1.641
# controls affected	0	0	3	2	1	0	1	5	3	1
*p*-value	1	1	8.6e-6	2.3e-8	1	0.55	1.3e-8	6.1e-5	7.6e-8	2.6e-5

Genes with expression levels greater than 1.5 times the wild type, or less than 2/3 the wild type, levels are considered to be affected by the knockout. The max change and average change rows represent the fold change in the expression level compared to the wild time. HS denotes that the yeast were heat shocked before extracting RNA. There were 20 total control genes. The p-value represents a Fisher’s exact score between the genes affected in the target and control groups.

Our first candidate regulator that we selected is *PBF2*, which is located in *cis* to eQTL hotspot V:350 kb and is known to regulate Ribi gene expression. Ribi genes are regulated transcriptionally through the *PAC* and *RRPE* promoter motifs, present upstream of most Ribi genes [27]. *PBF2* has previously been shown to bind to the *PAC* motif and to regulate Ribi gene expression in response to heat shock [22]. *PBF2* has nine missense mutations between the BY and RM strains and is differentially expressed between the strains that have the BY allele and those that have the RM allele (*p*-value = 3.0e-2). These mutations suggest that *PBF2* may function differently in the RM and BY strain, influencing Ribi gene expression directly. In our knock-out experiment, we did find that the target gene expression levels did increase dramatically for the *ΔPBF2* strain compared to the wild-type strain in response to heat shock. However, the change was negligible under normal growth conditions. Thus, our results again confirm that *PBF2* is involved in Ribi gene regulation.

We considered two candidate regulators from the VII:50 kb eQTL hotspot: *KAP114* and *RAI1*. *KAP114* is a nuclear importer that could have an indirect effect on Ribi gene transcription. *KAP114* is one of the 194 genes in all four eQTL modules. Nuclear import is an important step in Ribi; the Ribi proteins are translated in the cytoplasm and then *KAP* proteins import them into the nucleus, where these proteins assemble the RPs and rRNA into ribosomes [28]. Although *KAP114* has not yet been implicated in Ribi protein import, Ribi proteins and RPs account for most of the incoming nuclear traffic in a cell [28]. Therefore, if transport of these proteins into the nucleus was affected, the rate of Ribi transcription could be affected through a feedback loop. *RAI1* is a known Ribi gene; based on our results we hypothesize that *RAI1* affects the expression of other Ribi genes through a feedback loop or an unknown regulatory role. Our knock-out experiments found that predicted target genes were affected by the *ΔRAI1* strain in normal and in heat shock conditions. This is an interesting result as *RAI1* has no known regulatory role in Ribi gene expression. We found that the *ΔKAP114* strain had higher expression levels of many of the target genes (60%) during a heat shock, but that the expression of the targets was not affected under normal conditions. These results support the discovery of the V:50 kb hotspot as an important genomic region involved in the transcriptional regulation of Ribi genes.

GFlasso found associations from many SNPs on chromosome II to the Ribi genes. Thus, due to the large size of the eQTL hotspot on chromosome II:560 kb (about 200 kb), we had many candidate regulators to consider. Here we report an interesting mutation in the promoter region between *CNS1* and *RPB5*. *CNS1* and *RPB5* are both associated to all four eQTL hotspots in the Ribi group. There are seven SNPs and an indel in the promoter region, which could potentially affect the expression of both genes. *RPB5* is a component of RNA polymerase; a change in expression levels would directly perturb Ribi gene levels. Also, previous computational studies list *CNS1* as a possible Ribi regulator, although its involvement is unclear [8,9]. We also considered two other candidate regulators that we found: *SDS24* and *REI1*. *SDS24* is located near II:651 kb and *REI1* is located near II:739 kb; both locations are in the eQTL hotspot and are part of the eQTL module. In this experiment, we created knockout strains for *SDS24* and *REI1*, as *CNS1* and *RPB5* are essential genes. We did not find a significant difference in the target gene expression in the *ΔSDS24* strain. We did find, however, that the Ribi target genes were over-expressed in the *ΔREI1* knockout strain when compared to the wild type. This is another interesting observation, as *REI1* is a Ribi gene with no known regulatory role.

In summary, we have looked at three eQTL hotspots belonging to the Ribi group of hotspots and have shown experimentally that candidate regulators in each eQTL hotspot affect the expression of the target genes predicted by GFlasso. One interesting observation is that Ribi genes with no known regulatory role, such as *REI1* and *RAI1*, affected the expression of other Ribi genes (targets). This suggests that these Ribi genes might have an unknown regulatory role, or the association may uncover a feedback loop where other genes are over-expressed to compensate for the loss of other Ribi genes. On another note, out of the 43 targets predicted by GFlasso, only four genes were not affected by the regulators we tested, suggesting that GFlasso predicts the targets with high accuracy. Finally, it is interesting to note that all four validated regulators have a stronger effect in the case of heat shock, demonstrating the inconsistent effect that mutations can have in different growth environments.

### eQTL hotspots harbor mutations in NUP2, RIF2, and SIR3 that potentially affect telomere silencing

We now consider the four eQTL hotspots in the telomere group: IV:1500 kb, X:20 kb, XII:780 kb, and XII:1070 kb. Each of these eQTL hotspot are associated with the same 31 genes, and six additional genes are associated with three of the four eQTL hotspots. All 37 genes lie in telomere regions and three (IV:1500 kb, X:20 kb, and XII:1070 kb) of the four eQTL hotspots also lie in telomere regions. This suggests that there is coordinated or additive regulation among these four eQTL hotspots to turn on the expression of telomere genes. GO enrichment analysis found that these genes are enriched for the GO functional annotation "helicase activity" (*p*-value = 2e-17) and the GO component annotation "cellular component unknown" (*p*-value = 2e-17). This suggests that these genes share common function and interact with DNA as a helicase. Because many of the genes do not have a cellular component annotation, they might be understudied or not ordinarily expressed.

We considered the known function of each gene in the set individually and found seven yeast *YRF1* genes (*YRF1-1*, *YRF1-2*, *YRF1-3*, *YRF1-4*, *YRF1-5*, *YRF1-6*, and *YRF1-7*), see Table 5. We also found that the other 30 genes were all either "proteins of unknown function," or "helicase-like proteins encoded within the telomeric Y' element" (SGD database). Because the annotations were common across the genes in the set, we performed a sequence BLAST against *YRF1-1* and found that 36 of the 37 genes had high homology to the *YRF1-1* transcript (BLAST eValue < 1e-36). The 29 non-*YRF1* genes had no known functionality, despite their homology to the *YRF1* genes. We conclude that these genes are copies of *YRF1* in the yeast telomeres. The homology also suggests that these genes cross-hybridize to each other's probes on the microarray; if any one of the genes regulated by these four eQTL hotspots is expressed, all of the genes would appear to be expressed on the microarray. The homology of this module has been previously observed [9], however, in the RM wild type strain, the *YRF1* genes have a significantly lower expression level than in the BY mutant strain (*t*-test *p*-value = 1.1789e-26), which cannot be explained by the hypothesis of cross-hybridization. It appears that there is some kind of regulation turning on, or failing to turn off, at least one of the *YRF1* genes in the BY strain.

**Table 5:** Candidate regulators in the telomere group of eQTL hotspots

**eQTL Hotspot**	**Candidate**	**Differentially Expressed?**	**Mutations**	**Function**
**IV:1500 kb**	*YRF1-1*	6.4e-6	5 base insertion 186 nt upstream	*YRF1* gene
**X:20 kb**	*YJL225C*	1.1e-4	10 base insertion 237nt upstream	*YRF1*-like gene
**XII:780 kb**	*EST2*	No	5 missense	Telomerase component, reverse transcriptase
	*NUP2*	No	7 missense	Nuclear pore protein involved in telomere silencing
**XII:1070 kb**	*SIR3*	5.3e-3	12 missense	Involved in telomere silencing
	*RIF2*	No	6 missense	Involved in telomere silencing

In order to explain the difference in *YRF1* and *YRF1*-like gene expression between the RM and BY strains, we considered what is known about the *YRF1* genes. The *YRF1* genes are known to be a backup plan for telomerase. Telomerase is the protein complex essential for maintaining telomere length [29]. The loss of telomerase results in the gradual shortening of the telomeres and in eventual cell arrest, unless the telomeres are lengthened through the *YRF1* pathway [30]. There are many copies of *YRF1* located in yeast telomeres [31]. *YRF1* genes are not expressed in wild type cells, probably due to telomere silencing. However, it appears that as the telomeres shorten, silencing information is removed, leading to the expression of the *YRF1* genes [31]. The *YRF1* genes contain several helicase motifs and are believed to extend the telomeres through DNA homologous recombination, largely because other helicases participate in homologous recombination and genes important in homologous recombination are essential for survival without the proper function of telomerase [31].

Therefore, one possibility to explain the expression of *YRF1* is impaired telomerase function. Telomerase is made up of five proteins, two of which are functionally essential: *TLC1* and *EST2*[31]. *EST2*, a reverse transcriptase, is located in *cis* to the eQTL hotspot at XII:780 kb in the telomere group. We considered the sequence of the RM11-1a strain against the S288c strain and found five missense mutations in the *EST2* transcript (*EST2* was not differentially expressed between the two strains, *p*-value > .2), including an R to Q mutation in the reverse transcriptase domain. These mutations suggest that *EST2* could be impaired in its function, allowing for the shortening of telomeres and the activation of the *YRF1* genes. However, we conclude that the loss of *EST2* function is unlikely, given the popularity of the S288c strain and its use as a "normal" control for functional telomerase in yeast telomere studies [30]. We therefore suggest other pathways that potentially regulate *YRF1* gene expression.

Another possibility to explain the *YRF1* gene expression is the loss of telomere silencing genes. *NUP2* (XII:780 kb) and *RIF2*/*SIR3* (XII:1070 kb), are telomere silencing genes *cis* to hotspots in the telomere group. Dilworth et al. [32] report that *NUP2* (part of the nuclear pore complex) localizes in the nucleus with yeast telomeres; ChIP-chip experiments reveal that *NUP2* has a telomere binding preference. These results, combined with the association found by the GFlasso, suggest that *NUP2* (seven missense mutations) is a player in telomere silencing. In regards to *RIF2, RAP1* is also known to be involved in telomere silencing [33], and in this role it is assisted by *RIF2*; deletions in *RIF2* affect telomere length [34]. *SIR3* is also recruited to the telomere regions by *RAP1*, and cells lacking telomerase have increased concentration levels of the *SIR3* protein [30]. *SIR3* (twelve missense mutations) and *RIF2* (six missense mutations) are both located in *cis* to the XII:1070 kb eQTL hotspot. Previous computational analyses of this dataset have similarly implicated *RIF2* as a possible regulator of telomere genes [35].

A final possibility to explain the *YRF1* gene expression is the loss of silencing sequence in the DNA, leading to the expression of a *YRF1* transcript. *YRF1-1* (IV:1500 kb) and *YJL225C* (X:20 kb) both have mutations that could have this effect. *YRF1-1* has a five-base indel located 186 bases upstream in its promoter region, and *YJL225C* has a ten-base indel located 237 bases upstream in its promoter region. These mutations could potentially remove silencing information for these genes; the genotype at the eQTL hotspot is indicative of the expression level in both cases (*p*-value = 1e-4 and 1e-6 respectively).

In conclusion, we investigated three possibilities where mutations in the BY strain could lead to the expression of at least one *YRF1* gene transcript. We suggest that telomerase is not impaired in the BY strain, however, mutations in telomere silencing genes and in promoter regions of *YRF1* genes are likely candidates that may work together or in parallel to either turn on or silence *YRF1* gene expression.

### GFlasso uncovers 17 retrotransposon insertions

The retrotransposon group of eQTL hotspots are located on V:460 kb, VIII:110 kb, and XV:90 kb. The eQTL modules for each of these eQTL hotspots differ in size (42, 85, and 147 genes), although they all influence a common set of 20 genes, with 35 genes associated with two of the three eQTL hotspots. Although the VIII:110 kb eQTL module is enriched for conjugation (due to its close proximity with the mutated *GPA1* gene [17]), neither of the other two eQTL modules are enriched for a GO category. From our analysis, we found that the non-overlapping genes in these eQTL modules are not related to the genes involved in the overlap of the eQTL modules.

When we considered each of the 35 genes, we found that 15 of the genes are highly homologous (BLAST score of less than 1e-200 when queried against each other) and are annotated as Ty retrotransposons in the SGD database. We investigated the significance of finding 15 retrotransposons in the same eQTL module. A computational study [36] identified 331 retrotransposons in the yeast genome, 94 of which correspond to retrotransposon genes listed in the SGD. Of these 94 genes, 21 are included in the Brem and Kruglyak [13] dataset; it is unlikely that 15 of these 21 genes would end up in the same eQTL module of size 35 (*p*-value = 1.27e-30).

In yeast there are five types of retrotransposons, referred to as the Ty genes: Ty1, Ty2, Ty3, Ty4, and Ty5, with Ty1 being the most frequent in the genome [36]. Retrotransposons are scattered throughout the genomes of eukaryotes and function like a virus that is transcribed into an mRNA intermediate. This mRNA intermediate, through a reverse transcriptase, is then inserted back into the genome as cDNA, playing an important role in genome evolution [36,37]. It is estimated that Ty retrotransposon mRNA accounts for about 1% of the total mRNA in a cell. However an insertion into the chromosomal DNA only happens between 10^-7^ and 10^-8^ times per cell division cycle, suggesting that the insertion of the Ty genes is regulated post-transcriptionally [38,39]. The transcriptional regulation of the Ty genes happens through the TATA-box and other information in the promoter, and has been linked to the suppressor of transposition (*SPT*) genes and the *STE* genes [38].

Due to the sequence homology of these genes, it is probable that the observed co-expression is a result of cross-hybridization. However, we were interested to find genes in the three associated eQTL hotspots that could account for the transcriptional diversity between the strains. We found a few candidate *STP* and *STE* genes based solely on location, *SPT15* at V:464 kb and *STE20* at VIII:94 kb. However, sequence analysis revealed that *SPT15* is perfectly conserved between the RM and the BY strain. *STE20* had eight missense mutations and a few SNPs in its promoter region.

Interestingly, we found a retrotransposon located in *cis* to each of the three eQTL hotspots; each homologous to the 15 retrotransposons discovered in the eQTL module overlap. These three retrotransposons, *YER138C* (V:449 kb), *YHL009W-B* (VIII:85 kb), and *YOL104W-A* (XV:118 kb) are present in the S288c strain, but not in the RM strain sequence. This could be due to errors in the assembly of the RM sequence, but it likely that the insertion happened after these two strains diverged. We additionally considered each of the 15 Ty1 genes in the eQTL module overlap among these three contributing eQTL hotspots and found that only one Ty1 gene was present in both strains. Thus, we have found 17 total (14 in the dataset and 3 in *cis* to eQTL hotspots) retrotransposon insertions between the BY and RM strain, leaving open the possibility for other insertions as well. Additionally, among the 20 genes in the overlapping set of 35 that were not retrotransposons, we found that 13 of them were within 10 kb of a retrotransposon site and therefore could be expressed differently between the two strains because of the retrotransposon.

GFlasso has therefore uncovered a case where retrotransposon insertions have occurred since the BY and RM strain diverged. The occurrence of such retrotransposon insertion events in separate populations is not surprising and can be found by the direct comparison of the genome sequences of the two yeast strains. However, our analysis shows that in the absence of the full genome sequence information, GFlasso has the potential to discover systematic sequence differences such as gene insertions by investigating their impact on the expression levels solely based on an eQTL dataset.

## Discussion and conclusions

Many of the previous methods for discovering eQTLs from genotype and gene-expression datasets have been concerned with testing the hypothesis of association between an individual genotype and the expression of each gene [1]. However, there is a great deal of evidence that the elements in the genome and transcriptome interact with each other in performing a biological function; it has been widely recognized that the computational methods for detecting eQTLs should take into account this complex interaction pattern. Structured association mapping methods are powerful computational methods that directly map the quantitative-trait (gene-expression) network to genotypes, explicitly combining information across multiple correlated traits to increase the power of detecting association. There are a variety of structured association mapping methods available including others that consider gene networks, such as TreeLasso [40], in addition to other methods that consider population structure [41] or known information about the genome [42]. Indeed, it is important to take into account the inherent structures present in the data when looking for associations. Not only are there powerful methods available, but these methods are also available in a unified visualization software framework through GenAMap [16,43], that is easily assessable by analysts and experimentalists.

In this study, we re-analyzed the eQTL dataset [13] from the genetic cross of two yeast strains (BY and RM) using a structured association mapping method called GFlasso and discussed the new insights into yeast gene regulation that were provided by our analysis. The yeast eQTL dataset provides an excellent test-bed for comparing various computational methods, as it has been extensively analyzed. We showed that GFlasso, coupled with additional bioinformatics analysis, led to significant biological findings that had not been discovered by other methods, and we demonstrated the potential of GFlasso for future analyses of eQTL datasets that are becoming available for various organisms, tissue types, and diseases.

While the pleiotropic control of multiple genes by a genetic locus, called an eQTL hotspot, has been previously reported in analyses of many different eQTL datasets, our analysis of the yeast eQTL dataset revealed another layer of complexity in gene regulation by uncovering the pleiotropic effect of multiple genetic loci on multiple genes. The literature has yet to report this type of pleiotropic effects of multiple contributing genetic loci. Although our analysis in this study was focused on yeast, we suspect that the pleiotropic regulation of genes by multiple contributing eQTL hotspots is commonplace in many other eQTL datasets. Our results show that it may be worthwhile to revisit eQTL datasets with this new perspective, especially as more powerful computational methods become available. Furthermore, our results demonstrate the advantages of using structured association mapping in future studies to uncover weak signals and also of considering multiple genomic regions when identifying regulatory genes.

Our close investigation of the three groups of eQTL hotspots that control an overlapping set of genes led to new insights into Ribi gene regulation, telomere silencing, and retrotransposon activity and suggested potential regulators. By identifying missense and promoter mutations in the full DNA sequence of the candidate regulators, in addition to validation knock-out experiments, we provided strong evidence that these candidate regulators influence the gene expression levels of many genes in these biological pathways. In addition, we showed that prior studies of these individual genes in the literature support many of the hypotheses that the candidate regulators have the functional role suggested by our analysis. As yeast is one of the model organisms that have been studied extensively, a plethora of information is already available. This information includes the full genome sequence as well as detailed investigations of many of the genes; we were able to confirm the results of our analysis by comparing the results with this information. For many complex diseases, in other organisms where the same kind of extensive knowledge base is not yet available, we expect structured association mapping to serve as a powerful computational tool for new discoveries.

Our bioinformatics analysis of the three groups of eQTL hotspots opens up many research questions on the regulation of Ribi genes and telomere silencing that need to be further investigated in follow-up studies. Although our analysis suggests that the candidate regulators on different eQTL hotspots affect the same set of genes in a coordinated manner, understanding the exact mechanism of such coordination would require further research. For example, *NUP2* and *SIR3* on two different loci in the telomere group of eQTL hotspots have been found to regulate telomere silencing in both this analysis and previous studies of these genes. However, exactly how this interaction between these two genes occurs remains unexplored. This novel interaction could lead to further insight into how *NUP2* is involved in telomere silencing and perhaps uncover further interactions between various genetic loci that turn genes on and off.

Finally, GFlasso is designed to identify additive effects of multiple genetic loci on correlated traits, and thus, the effects of multiple eQTL hotspots on each gene were additive. An interesting future research direction would be to consider epistatic interactions among multiple eQTL hotspots, where the effect of a given eQTL hotspot is not independent of the genotypes of other eQTL hotspots. As detecting epistatic effects on individual genes is widely known as a computationally intensive task, the more challenging problem of detecting epistatic effects on multiple genes with pleiotropic effects would require a significant advance in computational tools.

## Methods

### Creating a network from gene expression data

GFlasso [7] takes a gene-interaction network as input, along with the genotype and gene-expression data, and performs a correlated association analysis to identify genomic regions that perturb correlated traits in the network. In order to obtain a gene-interaction network to use as an input to GFlasso, we used the algorithm for learning a topological overlap matrix as described in Zhang & Horvath [14] that was applied to the same dataset in Zhu et al. [9]. The resulting network has the properties of being modular and scale free with a few hub genes having high connectivity and controlling many other genes. Genes that appear correlated in this network often share common functionality and therefore are likely to be regulated by the same regions of the genome [9]. Complete details of our network construction method are available in Additional file 1: Supplementary Methods.

In order to improve the computational efficiency of running GFlasso, instead of performing a single GFlasso analysis on the full network of 5637 genes, we divided the full network into a set of smaller subnetworks, ran GFlasso on each subnetwork in parallel, and combined the results. We divided the full network into subnetworks so that the main connectivity structure with hub nodes and strong edges in the original network is preserved, while edges for weak correlation are ignored. We first identified connected components (four of size 16, 29, 45, and 3429) in the full network, where there are no edges going across different subnetworks. For the large connected component of size 3429, we ran a graph-clustering method called spectral clustering [44] to further divide it into eight smaller subnetworks. Complete details are provided in the Additional file 1: Supplementary Methods.

### GFlasso analysis

GFlasso is a sparse multivariate regression method that we have previously developed for finding a correlated genome association for multiple related traits given a trait network and genotype/phenotype dataset [7]. GFlasso extends the standard lasso [45] that uses an *L*_*1*_ penalization to shrink the regression coefficients (or parameters for association strengths) towards zero and obtain a sparse estimate with many zero-valued coefficients for SNPs with no associations. The lasso has been widely used for single-trait association analysis. In addition to the lasso penalty, GFlasso introduces another penalty function, called a graph-guided fused-lasso penalty, which is constructed from the trait network and plays the role of enforcing a soft constraint that highly correlated traits in the network are influenced by a common region in the genome. Given an *N* x *J* genotype data matrix *X*, where *N* is the number of strains and *J* is the number of SNPs, and an *N* x *K* gene-expression data matrix *Y*, where *K* is the number of genes, GFlasso estimates the regression coefficients *B*, a *J* x *K* matrix, for association strengths by solving the following optimization problem:

$$\begin{array}{cc} \;B=\mathrm{argmin} & \sum {}_{k}{\left(y_{k}-X\beta_{k}\right)}^{T}\left(y_{k}-X\beta_{k}\right)+\lambda \sum {}_{k}\sum {}_{j}\left|\beta_{\mathrm{jk}}\right|+\gamma \\ & \times \sum {{}_{\left(m,l\right)\in }}_{E}f\left(r_{\mathrm{ml}}\right)\sum {}_{j}\left|\beta_{\mathrm{jm}}-\mathrm{sign}\left(r_{\mathrm{ml}}\right)\beta_{\mathrm{jl}}\right|, \end{array}$$

where *y*_*k*_ and *β*_*k*_ denote the *k*th column of *Y* and *B*. The last term in the above equation is the graph-guided fused-lasso penalty that encourages two association strengths *β*_*jm*_ and *β*_*jl*_ to have similar values if the *m*th and *l*th traits are correlated and connected with an edge (*m,l*)Є*E.*

The *λ* and *γ* in the above equation are the regularization parameters that control the amount of penalization. We found the optimal values for *λ* and *γ* through cross-validation as follows. We divided the full dataset into one training set (104 strains) and one validation set (10 strains), ran GFlasso on the training set for different values of *λ* and *γ*, and computed prediction error on the validation set. This was done on one split of the data only due to the computation running time of the algorithm. We selected the values of *λ* and *γ* with the lowest validation-set error as the optimal values. Instead of performing a grid search over *λ* and *γ*, we first fixed *γ* = 0 and searched for an optimal value of *λ* on the log10 scale. The *λ* with the smallest prediction error is then used to search for an optimal value of *γ* on the log10 scale. Then, we ran a fine-tuned search for *λ* given *γ*. Once we obtained the optimal estimate of association strengths *B*, we considered all SNP/gene pairs corresponding to non-zero entries in *B* as significantly associated.

### GenAMap

We developed a software platform called GenAMap [15,16] that implements the full analysis pipeline of structured genome-phenome association analysis and provides tools to visualize the results from the analysis. The GFlasso analysis pipeline that we used in this paper is fully integrated into GenAMap, including creating the trait network and running the GFlasso program on the eQTL data. GenAMap presents the user with interactive visualizations that show the structure of the genome and the phenome when browsing association results. GenAMap is a visual analytic tool for a variety of other spare-structured regression techniques for structured genome-phenome associations [40,41]. GenAMap also helps to scale the computational running time of these algorithms (running time of various algorithms, including GFlasso is discussed in our previous work [7,43]).

### Identifying mutations between the strains

In order to identify the genotypic differences in genes across two strains, we downloaded the RM11-1a sequence for each protein of interest from the *Saccharomyces cerevisiae* RM11-1a sequencing project website [46] and used it as the query for BLASTp search [47], limiting the results to the *Saccharomyces cerevisiae* S288c strain. The full protein sequences that we obtained as results for each query were used to identify mutations between the two strains. In the cases of promoter mutations, we took the 500 bases upstream of the gene in the RM sequence and performed a BLASTn search [47] in the BY strain.

### Preparing knock-out yeast strains

Knockout strains of yeast were obtained from Open Biosystems. Overnight cultures of yeast grown in YEPD medium at 25°C were diluted ten-fold into 25 ml of YEPD and grown at 25°C to 5 × 10^8^ cells/ml. Then, 12.5 ml of the culture was grown for another 15 min at 25°C, and 12.5 ml was shifted to 38° C for 15 min. Cells were harvested by centrifugation, washed once in sterile water, and cell pellets were frozen.

### Measuring the expression levels

To measure the expression levels of the selected genes in the wildtype and the five knockout mutant strains, we performed nanoString expression analysis. Total RNA was extracted using the Qiagen RNeasy Plant kit (Cat #74904). 800 ng of total RNA was mixed with the nanoString probe set and incubated at 65°C overnight (12-18 hours). The reaction mix was then loaded on the nanoString nCounter Prep Station for binding and washing, using the default program. The resultant cartridge was then transferred to the nanoString nCounter digital analyzer for scanning and data collection. A total of 600 fields were captured per sample. The raw data, in a form of digital counts for each of the probe target genes in every sample, were first adjusted for binding efficiency and background subtraction using the manufacturer included positive and negative controls, following nCounter data analysis guidelines. Second, mutant strain data sets were normalized to the control wildtype strain using the 20 included control genes. The normalized data sets were used to determine if the expression level of a gene in a mutant was different from that in the wild-type control. Results were visualized using Heatmap builder [48].

## Competing interests

REC and EPX have filed a patent application for GenAMap, the software used to conduct this analysis.

## Authors’ contribution

REC carried out the computational analysis and drafted the manuscript. SK provided key insight throughout the analysis and drafted the manuscript. JLW provided expert advice and helped coordinate the molecular experiments. WX performed the nanostring experiments. EPX directed the project and drafted the manuscript. All authors read and approved the final manuscript.

## Supplementary Material

Additional file 1 Supplementary Methods. Click here for file

Additional file 2 Supplementary GFlasso results. Click here for file

Additional file 3 Supplementary knockout experiment results. Click here for file

## References

[B1] Gilad Y Rifkin SA Pritchard JK Revealing the architecture of gene regulation: the promise of eQTL studies Trends Genet 2008 24 8 408 445 10.1016/j.tig.2008.06.001 18597885 PMC2583071

[B2] Brem RB Yvert G Clinton R Kruglyak L Genetic dissection of transcriptional regulation in budding yeast Science 2002 296 5568 752 755 10.1126/science.1069516 11923494

[B3] Chen Y Zhu J Lum PY Yang X Pinto S Variations in DNA elucidate molecular networks that cause disease Nature 2008 452 429 435 10.1038/nature06757 18344982 PMC2841398

[B4] Stranger BE Nica AC Forrest MS Dimas A Bird CP Population genomics of human gene expression Nat Genet 2007 39 1217 1224 10.1038/ng2142 17873874 PMC2683249

[B5] West MAL Kim K Kliebenstein DJ van Leeuwen H Michelmore RW Global eQTL mapping reveals the complex genetic architecture of transcript-level variation in Arabidopsis Genetics 2007 175 3 1441 1450 17179097 10.1534/genetics.106.064972 PMC1840073

[B6] Yaguchi H Togawa K Moritani M Itakura M Identification of candidate genes in the type 2 diabetes modifier locus using expression QTL Genomics 2005 85 5 591 599 10.1016/j.ygeno.2005.01.006 15820311

[B7] Kim S Xing EP Statistical estimation of correlated genome associations to a quantitative trait network PLoS Genet 2009 5 8 e1000587 10.1371/journal.pgen.1000587 19680538 PMC2719086

[B8] Lee SI Dudley AM Drubin D Silver PA Krogan NJ Learning a prior on regulatory potential from eQTL data PLoS Genet 2009 5 1 e1000358 10.1371/journal.pgen.1000358 19180192 PMC2627940

[B9] Zhu J Zhang B Smith EN Drees B Brem RB Integrating large-scale functional genomic data to dissect the complexity of yeast regulatory networks Nat Genet 2008 40 7 854 861 10.1038/ng.167 18552845 PMC2573859

[B10] Leonardo B Enrico P Stefan B François C StuartA C Bayesian Detection of Expression Quantitative Trait Loci Hot Spots Genetics 2011 189 4 1449 1459 10.1534/genetics.111.131425 21926303 PMC3241411

[B11] Lei B Xuefeng X Yan C Expression QTL Modules as Functional Components Underlying Higher-Order Phenotypes PLoS One 2010 5 12 e14313 10.1371/journal.pone.0014313 21179437 PMC3001472

[B12] Wei Z Jun Z Schadt EE Liu JS A Bayesian Partition Method for Detecting Pleiotropic and Epistatic eQTL Modules PLOS Comp Bio 2010 6 1 e1000642 10.1371/journal.pcbi.1000642 PMC2797600 20090830

[B13] Brem RB Kruglyak L The landscape of genetic complexity across 5700 gene expression traits in yeast Proc Natl Acad Sci USA 2005 102 5 1572 1577 10.1073/pnas.0408709102 15659551 PMC547855

[B14] Zhang B Horvath S A General Framework for Weighted Gene Co-Expression Newtork Analysis Stat Appl Genet Molec Biol 2005 4 1 Article 17 10.2202/1544-6115.1128 16646834

[B15] Curtis RE Xing EP GenAMap: An Integrated Analytic and Visualization Platform for GWA and eQTL Analysis Proceedings of the 17th International Conference on Intelligent Systems for Molecular Biology (ISMB) 2010 Technology Track

[B16] Curtis RE Kinnaird P Xing EP GenAMap: visualization strategies for association mapping IEEE Symposium on Biological Data Visualization 2011 1 87 95

[B17] Yvert G Brem RB Whittle J Akey JM Foss E Trans-acting regulatory variation in Saccharomyces cerevisiae and the role of transcription factors Nat Genet 2003 35 57 64 12897782 10.1038/ng1222

[B18] The Saccaromyces Genome Database [Online]. http://yeastgenome.org

[B19] Chua G Morris QD Sopko R Robinson MD Ryan O Identifying transcription factor functions and targets by phenotypic activiation PNAS 2006 103 32 12045 12050 10.1073/pnas.0605140103 16880382 PMC1567694

[B20] Hughes TR Marton MJ Jones AR Roberts CJ Stoughton R Functional discovery via a compendium of expression profiles Cell 2000 102 1 109 126 10.1016/S0092-8674(00)00015-5 10929718

[B21] MacIssac KD Wang T Gordon DB Gifford DK Stormo GD An improved map of conserved regulatory sites for Saccharomyces cerevisiae BMC Bioinformatics 2006 7 113 10.1186/1471-2105-7-113 16522208 PMC1435934

[B22] Zhu C Byers KJ McCord RP Shi Z Berger MF High-resolution DNA-binding specificity analysis of yeast transcription factors Genome Res 2009 19 4 556 566 10.1101/gr.090233.108 19158363 PMC2665775

[B23] Harbison CT Gordon DB Lee TI Rinaldi JN MacIssac KD Transcriptional regulatory code of a eukaryotic genome Nature 2004 431 99 104 10.1038/nature02800 15343339 PMC3006441

[B24] Lee TI Rinaldi NJ Robert F Odom DT Bar-Joseph Z Transcriptional Regulatory Networks in Saccharomyces cerevisiae Science 2002 298 5594 799 804 10.1126/science.1075090 12399584

[B25] Brem RB Storey JD Whittle J Kruglyak L Genetic interactions between polymorphisms that affect gene expression in yeast Nature 2005 436 7051 701 703 10.1038/nature03865 16079846 PMC1409747

[B26] Wei Z Jun Z EricE S JunS L A Bayesian Partition Method for Detecting Pleiotropic and Epistatic eQTL Modules PLoS Comp Bio 2010 6 1 e1000642 10.1371/journal.pcbi.1000642 PMC2797600 20090830

[B27] Hughes JD Estep PW Tavazoie S Church GM Computational identification of cis-regulatory elements associated with groups of functionally related genes in Saccharomyces cerevisiae J Mol Biol 2000 296 5 1205 1214 10.1006/jmbi.2000.3519 10698627

[B28] Sydorskyy Y Dilworth DJ Yi EC Goodlett DR Wozniak RW Intersection of the Kap123p-mediated nuclear import and ribosome export pathways Mol Cell Biol 2003 23 6 2042 2054 10.1128/MCB.23.6.2042-2054.2003 12612077 PMC149464

[B29] Cohn M Blackburn EH Telomerase in yeast Science 1995 269 5222 396 400 10.1126/science.7618104 7618104

[B30] Straatman KR Louis EJ Localization of telomeres and telomere-association proteins in telomerase-negative Saccharomyces cerevisiae Chromosome Res 2007 15 1033 1050 10.1007/s10577-007-1178-2 18075778 PMC2784495

[B31] Yamada M Hayatsu N Matsuura A Ishikawa F Y'-Help1, a DNA helicase encoded by the yeast subtelomeric Y' element is induced in survivors defective for telomerase J Biol Chem 1998 273 50 33360 33366 10.1074/jbc.273.50.33360 9837911

[B32] Dilworth DJ Tackett AJ Rogers RS Yi EC Christmas RH The mobile nucleoporin Nup2p and chromatin-bound Prp20p function in endogenous NPC-mediated transcriptional control J Cell Biol 2005 171 6 955 965 10.1083/jcb.200509061 16365162 PMC2171315

[B33] Shore D Telomere length regulation: getting the measure of chromosome ends Biol Chem 1997 387 7 591 597 9278138

[B34] Teixeira MT Arneric M Sperisen P Lingner J Telomere length homeostasis is achieved via a switch between telomerase- extendable and -nonextendable states Cell 2004 117 3 323 325 10.1016/S0092-8674(04)00334-4 15109493

[B35] Lee SI Pe'er D Dudley AM Church GM Koller D Identifying regulator mechanisms using individual variation reveals key role for chromatin modification PNAS 2006 103 38 14062 14067 10.1073/pnas.0601852103 16968785 PMC1599912

[B36] Kim JM Vanguri S Boeke JD Gabriel A Voytas DF Transposable elements and genome organization: a comprehensive survery of retrotransposons revealed by the complete Sacchararomyces cerevisiae genome sequence Genome Res 1998 8 5 464 478 9582191 10.1101/gr.8.5.464

[B37] Boeke JD Sandmeyer SB Broach JR, Jones EW, Pringle J "Yeast transposable elements," The molecular and cellular biology of the yeast Saccharomyces: genome dynamics, protein synthesis, and energetics 1991 1 Cold Spring Harbor, New York: Cold Spring Harbor Laboratory Press 193 261

[B38] Krastanova O Hadzhitodorov M Pesheva M Ty Elements of the yeast Saccharomyces cerevisiae Biotchnol Biotc Eq 2005 19 3 19 26

[B39] Curcio MJ Hedge AM Bocke JD Garfinkel DJ Ty RNA levels determine the spectrum of retrotranposition events that activate gene expression in Saccharomyces cerevisiae Mol Gen Genet 1990 220 213 221 2157950 10.1007/BF00260484

[B40] Kim S Xing EP Tree-guided group lasso for multi-task regression with structured sparsity Proceedings of the 27th International Conference on Machine Learning (ICML) 2010

[B41] Puniyani K Kim S Xing EP Multi-population GWA mapping via multi-taks regularized regression Bioinformatics 2010 26 12 i208 i216 10.1093/bioinformatics/btq191 20529908 PMC2881376

[B42] Lee S Zhu J Xing EP Adaptive Multi-Task Lasso: with Application to eQTL Detection Advances in Neural Information Processing Systems 23 (NIPS) 2010 [http://www.proceedings.com/10901.html]

[B43] Curtis RE Goyal A Xing EP Enhancing the usability and performance of structured association mapping algorithms using automation, parallelization, and visualization in the GenAMap software system BMC Genet 2012 13 24 10.1186/1471-2156-13-24 PMC3342145 22471660

[B44] Luxburg U A tutorial on spectral clustering Statistics and Computing 2007 17 4 395 416 10.1007/s11222-007-9033-z

[B45] Tibshirani R Regression shrinkage and selection via the lasso Royal Statist Soc B 1996 58 1 267 288

[B46] Saccharomyces cerevisiae RM11-1a sequencing project [Online] http://www.broadinstitute.org/annotation/genome/saccharomyces_cerevisiae/Home.html

[B47] NCBI [Online] http://blast.ncbi.nlm.nih.gov/Blast.cgi

[B48] King JY Ferrara R Tabibiazar R SpinJ M Chen MM Pathway analysis or coronary atherosclerosis Physiol Genomics 2005 23 1 103 118 10.1152/physiolgenomics.00101.2005 15942018

