# Supplementary material for: Structured association analysis leads to insight into Saccharomyces cerevisiae gene regulation by finding multiple contributing eQTL hotspots associated with functional gene modules
Source: BMC Genomics. 2013 Mar 21;14:196. doi: 10.1186/1471-2164-14-196 (PMC3616858; doi:10.1186/1471-2164-14-196)
Supplement: Additional file 1 — Supplementary Methods. [file 1471-2164-14-196-S1.doc]

# GFlasso analysis leads to insight into *Saccharomyces cerevisiae* gene regulation by uncovering interacting eQTL hotspots

RE Curtis, S Kim, JL Woolford, EP Xing

Contents

# Supplementary Figures


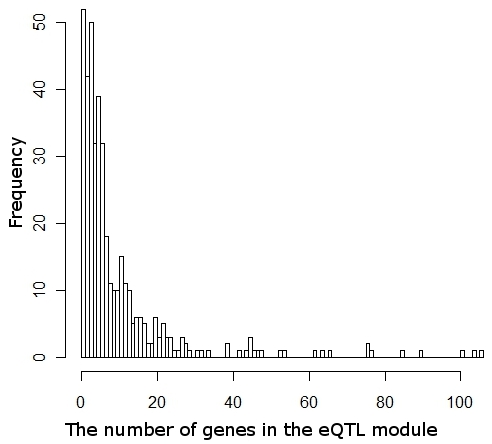


Supplementary Figure 1: Histogram of the number of genes in each of the 428 eQTL modules. We considered the genomic regions corresponding to eQTL modules with more than 40 genes as eQTL hotspots. The largest 13 eQTL modules (with more than 110 genes per eQTL module) are not shown.


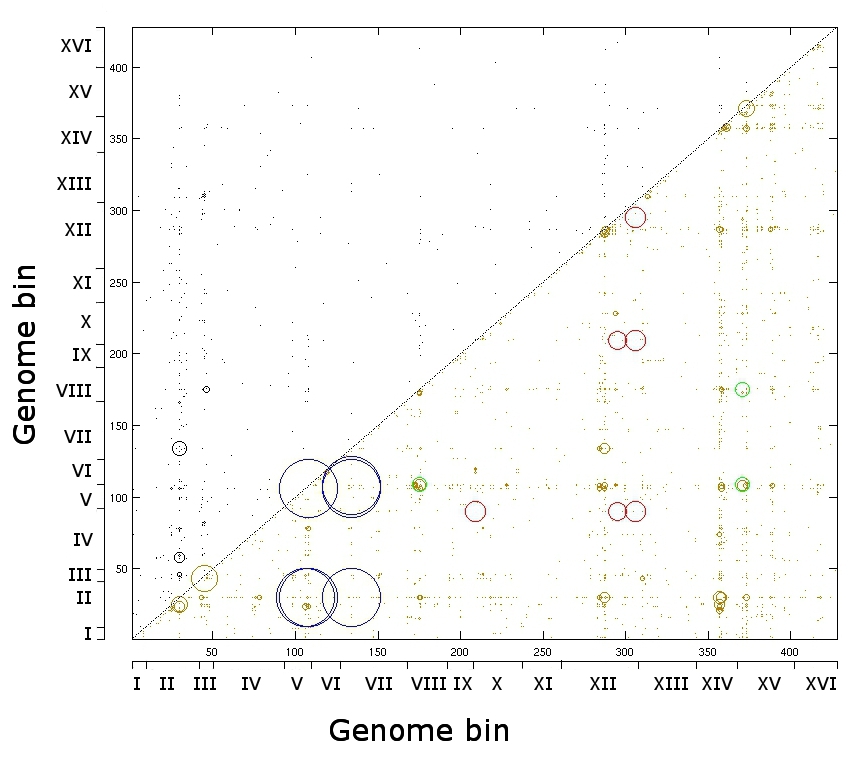


Supplementary Figure 2: Comparison of interacting genome locations found by GFlasso with epistatically interacting SNPs found by an analysis using a previously described non-parametric method [ HYPERLINK "" \l "Bre051" 1 ]. The results from the nonparametric method are shown on the upper diagonal, and the GFlasso results are shown on the lower diagonal. The size of the circle is proportional to the number of genes in the overlap of the two eQTL modules of the two hotspots. The interactions reported in the main text are colored in blue for the Ribi group, red for the telomere group, and green for the Retrotransposon group. Each genome bin corresponds to one of the 428 eQTL modules found by GFlasso.


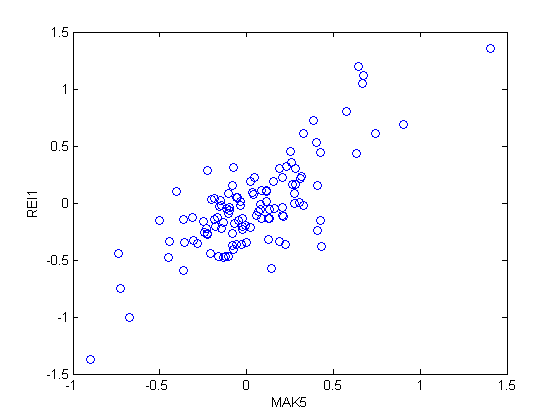


(a)


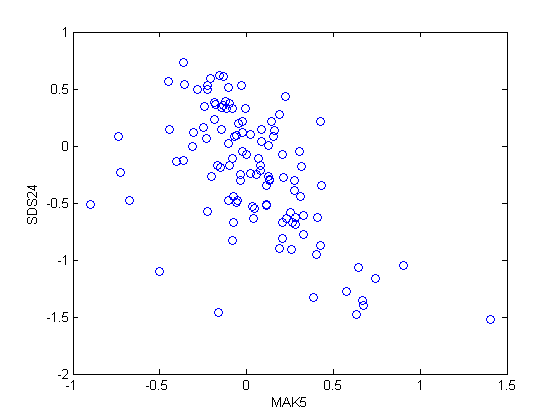


(b)


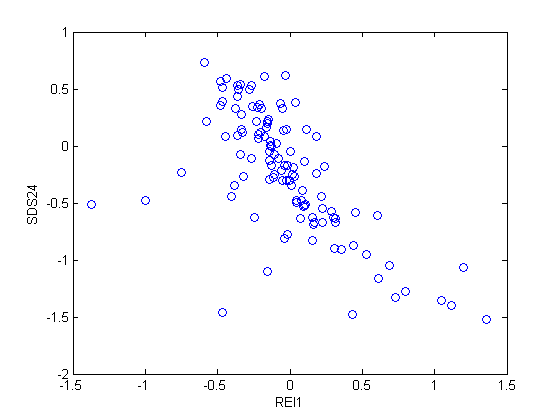


(c)

Supplementary Figure 3: Correlation of gene expression among MAK5, REI1, and SDS24. All of the three genes are located in *cis* to eQTL hotspot II:560kb. MAK5 is located in II:528kb, REI1 in II:740kb, and SDS24 in II:651kb. (a) MAK5 is positively correlated with REI1 (*r*^2^ = 0.7719). (b) MAK5 is negatively correlated with SDS24 (*r*^2^ = -0.6569). (c) REI1 is negatively correlated with SDS24 (*r*^2^ = -0.5680)

# Supplementary Methods

## Preprocessing yeast eQTL dataset

We used the expression and gene expression data collected from 112 segregants (114 strains) from a cross between BY4716 and RM11-1a 2]. The dataset has 2956 SNPs across the 16 chromosomes of the yeast genome. Additionally, for each segregant, microarray expression data are available for 6216 genes. After pre-processing the microarray data with standard procedures, we discarded the genes whose expression values are missing for more than 30% of the strains, and used the gene expression values for the other 5637 genes in our GFlasso analysis. We imputed the missing values for these genes using *k*-nearest-neighbor imputation method [ HYPERLINK "" \l "Tro01" 3 ].

In the genotype data, we found that many of the adjacent SNPs are in complete linkage disequilibrium (LD) across all 114 strains. After discarding those adjacent SNPs with perfect correlations, we were left with 1260 SNPs, which we used in our GFlasso analysis.

## Learning the gene-interaction network

We applied the soft-thresholding methodology 4] to gene expression data to learn the gene interaction network that can be used in GFlasso [ HYPERLINK "" \l "Kim09" 5 ]. Below, we provide the details of the procedure for network generation.

1. We computed a correlation matrix of size 5637x5637 for 5637 genes, and took the absolute values of its elements.
2. For different values of *η* ranging from 1 to 20, we raised each value in the matrix element-wise to the *η* power and obtained 20 different matrices. For each of the 20 matrices, we perform the following procedure. We regressed log(*p*(*k*)) on log(*k*) to find *r*^2^, where *k* is the number of neighbors (degree) of each gene (calculated as the sum over all columns for each row for the gene of the matrix), and *p*(*k*) is the degree distribution of the network. *p*(*k*) for each gene was found empirically by creating a 50-bin histogram for all genes in the network based on *k,* and then dividing the number of genes in the same bin as the gene in question by the total number of genes.
3. Out of the 20 networks, we selected the scale-free network corresponding to the lowest value of *η* that gives *r*^2^>.8. This was obtained at *η* =5, which is consistent with the previous reports from using the same method on these data6]. Similar to the previous application of this method, we found that the network degree distribution is described as *p*(*k*)~ *k*^1.47^ and *r*^2^=.84.
4. We used the scale-free network to create a topological overlap matrix (TOM). After setting all diagonal elements to zero, a TOM was found by calculating *ω* for each element in the matrix as previously described [ HYPERLINK "" \l "Zha051" 4 ]. We multiplied each entry in the TOM by its original sign in the correlation matrix to preserve positive and negative edge weights.
5. We discarded edges for weak interactions by thresholding the TOM at 0.10, since these weak edges only add to the computational cost of GFlasso without increasing the power of detecting association.

## Clustering the gene interaction network

Instead of using the full gene interaction network in GFlasso, in order to reduce the computational cost, we used connected component analysis and spectral clustering to cluster the full network into ten smaller subnetworks (of size around 650 genes) that are computationally manageable by GFlasso.

### Finding connected components

If there are no edges present between sets of genes in the gene interaction network, performing GFlasso analysis on each of the connected components separately and combining the results would be equivalent to running GFlasso on the full network. Our connected component analysis found 2201 genes that were not connected to any other genes, and connected components of size 16, 29, 45, and 3249. All of these connected components were small enough for GFlasso to handle except for the one with 3429 genes. As we discuss in the next section, we used the spectral clustering algorithm on this large connected component of size 3429 to further cluster it into smaller subnetworks.

### Running the spectral clustering algorithm

We used the spectral clustering algorithm as implemented in Chen et al 7] to further cluster the large connected component of 3429 genes. Spectral clustering requires the user to pre-define the number of clusters, and we selected the results with eight clusters, since all of the eight clusters contained fewer than 650 genes, which is a computationally manageable size for GFlasso. The largest of the eight clusters contained 610 genes. By running spectral clustering algorithm, we lose information on relatively weaker correlations among genes, but preserve the most connected parts of the network.

Once we have the connected components and the clusters from spectral clustering, we group the different clustering into ten subnetworks of about 650 genes.

## Initializing the GFlasso analysis

For GFlasso analysis, we initialized the regression coefficients using the results from the lasso. We used the fast implementation of the lasso available as an R package, glmnet [ HYPERLINK "" \l "Fri" 8 ]. We ran glmnet on the expression values of each gene and all 1260 SNPs, and combined the results into a single matrix of regression coefficients.

In addition to the regression coefficients for association strengths, the lasso has the regularization parameter *λ* whose value needs to be determined. We used the validation-set error to find the optimal value for *λ* as follows. We divided the dataset into a training set of 104 strains and a validation set of 10 strains, and ran lasso on the training set for different values of *λ*. Then, we calculated the prediction error using the validation set and the estimate of regression coefficients from the training set, and selected the value of *λ* with the smallest validation-set error as the optimal *λ*. Using this procedure, we performed an initial search for the optimal *λ,* for the range of *λ* from *λ*=1e-4 to *λ*=1.64 increasing by the log2 scale. The smallest validation-set error was obtained at *λ*=0.1. Then, we performed a refined search for the optimal *λ* with a range of *λ* from *λ*=.01 to *λ=*.2 with step size of 0.01. The *λ* with the smallest validation-set error was obtained at 0.11. The lasso estimate of the regression coefficients obtained at *λ*=.11 was used to initialize all of our GFlasso runs.

## Procuring the GFlasso results

We used the version of GFlasso in C++ that is available online (<http://sailing.cs.cmu.edu/gflasso>). GFlasso has two regularization parameters *λ* and *γ*, and we use a three-stage process to find values for *λ* and *γ*.

### Finding the optimal value for *λ* given *γ=*0

In the first stage, we used validation-set error to determine the value for *λ* with *γ* fixed at 0. We ran GFlasso on the training set with *λ* set to different values, 1e-5, 1e-4, 1e-3, 1e-2, 1e-1, 1e0, and 1e1, computed the prediction error using the validation set and the estimate of regression coefficients from the training set, and then selected the *λ* with the smallest validation-set error as optimal given *γ=*0. In each run of GFlasso, we ran the algorithm on each subnetwork of genes found by connected component analysis and spectral clustering algorithm as described in previous sections, and combined the results for different subnetworks to compute the validation-set error.

As GFlasso sets the regression coefficients of the non-association SNPs to near-zero values rather than exact zeros, we used 1e-5 as a threshold to set the near-zero entries of the regression coefficient matrix to exact zeros. Following the standard procedure for penalized regression, after each run of GFlasso on the training data, we re-estimated the values of the non-zero elements in the regression coefficient matrix using the least-squared-error method, and then calculated the prediction error. The smallest prediction error given *γ=*0 was found at *λ*=1.0.

### Finding the optimal value for *γ* given *λ*=1*.*0

In the second stage, we set *λ*=1.0 which was obtained in the first stage, and used the similar procedure as described in the previous section to search for the optimal value for *γ.* We ran GFlasso with *γ* = 1e-5, 1e-4, 1e-3, 1e-2, 1e-1, 1e0, and 1e1, and found that the smallest validation-set error was obtained at *γ*=1e-3.

### Obtaining the final estimate of the parameters

In the last stage, we set *γ* =1e-3 using the estimate from the second stage and performed a search for the optimal value for *λ* using the range of *λ* from 0 to 1 with the step size of .01. We found that the smallest validation-set error given *γ* =1e-3 was obtained at *λ*=.72.

## SNP LD analysis

In order to interpret the results from GFlasso, we group SNPs in high LD into bins, and group the association results from GFlasso analysis according to these SNP bins. In other words, within each bin of SNPs in high LD, we treat the associations from each member SNP to any phenotypes as an association to the common genomic locus.

We applied the greedy algorithm for tag SNP selection to group the SNPs [ HYPERLINK "" \l "Car04" 9 ]. We first created a matrix of size 1260x1260, whose element contains the *r*^2^ LD statistic between SNPs, and set all elements with *r*^2^<0.80 to zero, since we considered only those SNP pairs with *r*^2^>0.80 as in high LD. Then, we applied the following iterative procedure.

1. Find the pair of SNPs with the highest LD by selecting the element with the highest value in the matrix. We call the selected two SNPs “founders”.
2. Combine the founder SNPs and the SNPs located between the founder SNPs to create a new bin.
3. Expand the bin in Step 2) by adding all of adjacent SNPs that are in high LD (*r*^2^ > 0.8) with the founder SNPs.
4. Repeat Steps 1)-3) until all SNPs have been assigned to a bin or form a bin of a single SNP with no significant LD with other SNPs.

## eQTL Module creation and analysis

We created eQTL modules for each of the 428 bins by finding all genes in the ***B*** matrix that had a nonzero value for any SNP in the bin. We then picked the largest, most interesting bins for further analysis. We left out contiguous bins that were mapped to the same genes as a neighboring bin we had already chosen. For bins with eQTL modules larger than 40 genes, we call them *eQTL hotspots*.

For each bin, we performed enrichment tests using the GO Slim annotation ( HYPERLINK "http://www.yeastgenome.org/" http://www.yeastgenome.org/). We also performed enrichment tests using two knockout datasets and four transcription factor (TF) binding datasets. We used Fisher’s exact test to perform all of our enrichment calculations. The enrichment scores for all 428 eQTL modules with respect to GO category are reported in the SupplementaryWorksheet.xls.

We performed knock-out enrichment analyses using two knockout datasets. The first knockout data set that we used is the yeast compendium data set . We followed the procedure used by Zhu et al (2008) to preprocess the data. The second knockout data set that we used is from Chua et al . In our preprocessing of this dataset, we considered a gene to be affected by a knockout if its *z*-score was greater than 2.58 or less than -2.58.

We used four different TF datasets to test for TF enrichment. The first data that we used was a ChIP-on-chip dataset . We used the results from this dataset that corresponded to a threshold of .005 and a stringent cutoff. The second dataset that we used is from Lee et al , and the third data set is from Harbison et al . For both of these datasets we used the conservative threshold of .001 to indicate a binding preference. Finally, we performed enrichment tests using the Protein Binding array results from the Supplementary material of Zhu et al. . We used the top 200 genes reported for each TF for our enrichment analysis.

The results from each enrichment analysis on each of the 428 eQTL modules are listed in the SupplementaryWorksheet.xls. We found that the same patterns in the TF and knockout datasets that we found in the GO enrichment analysis. eQTL modules that overlapped were enriched for binding by the same TFs, or enriched for perturbation by the same knockout genes. eQTL modules mapping to eQTL hotspots were not just enriched for common function (GO category), but were also enriched for TF binding to a particular factor and/or knockout-signature of a particular gene.

# References
